# HSPD1 Supports Osteosarcoma Progression through Stabilizing ATP5A1 and thus Activation of AKT/mTOR Signaling

**DOI:** 10.7150/ijbs.100015

**Published:** 2024-09-23

**Authors:** Yiming Zhang, Ruilin Pan, Kun Li, Lek Hang Cheang, Jing Zhao, Zhangfeng Zhong, Shaoping Li, Jinghao Wang, Xiaofang Zhang, Yanmei Cheng, Xiaofei Zheng, Rongrong He, Huajun Wang

**Affiliations:** 1 Department of Sports Medicine, The First Affiliated Hospital, Guangdong Provincial Key Laboratory of Speed Capability, The Guangzhou Key Laboratory of Precision Orthopedics and Regenerative Medicine, Jinan University, Guangzhou, China.; 2 State Key Laboratory of Bioactive Molecules and Drug Ability Assessment, Guangdong Engineering Research Center of Chinese Medicine & Disease Susceptibility, International Cooperative Laboratory of Traditional Chinese Medicine Modernization and Innovative Drug Development of the Chinese Ministry of Education, Guangdong Province Key Laboratory of Pharmacodynamic Constituents of Traditional Chinese Medicine and New Drugs Research, Jinan University, Guangzhou, China.; 3 Department of Orthopedic Surgery, Centro Hospitalar Conde de Sao Januario, Macau, China.; 4 State Key Laboratory of Quality Research in Chinese Medicine, Institute of Chinese Medical Sciences, Department of Pharmaceutical Sciences, Faculty of Health Sciences, University of Macau, China.; 5 Department of Pharmacy, the First Affiliated Hospital, State Key Laboratory of Frigid Zone Cardiovascular Diseases, Jinan University, Guangzhou, China.; 6 Department of Orthopedics, NHC Key Laboratory of Cell Transplantation, The First Affiliated Hospital of Harbin Medical University, Harbin, China.; 7 Department of Pharmacology (State-Province Key Laboratories of Biomedicine-Pharmaceutics of China, Key Laboratory of Cardiovascular Research, Ministry of Education), College of Pharmacy, Harbin Medical University, Harbin, 150086, Heilongjiang, China.; 8 Department of Cardiothoracic Surgery ICU, The First Affiliated Hospital of Sun Yat-sen University, Guangzhou, 510080, Guangdong, China.

**Keywords:** osteosarcoma, multiomics analysis, HSPD1, ATP5A1, ubiquitination, oxidative phosphorylation

## Abstract

Malignant transformation is concomitant with excessive activation of stress response pathways. Heat shock proteins (HSPs) are stress-inducible proteins that play a role in folding and processing proteins, contributing to the non-oncogene addiction of stressed tumor cells. However, the detailed role of the HSP family in osteosarcoma has not been investigated. Bulk and single-cell transcriptomic data from the GEO and TARGET databases were used to identify HSPs associated with prognosis in osteosarcoma patients. The expression level of HSPD1 was markedly increased in osteosarcoma, correlating with a negative prognosis. Through *in vitro* and *in vivo* experiments, we systematically identified HSPD1 as an important contributor to the regulation of proliferation, metastasis, and apoptosis in osteosarcoma by promoting the epithelial-mesenchymal transition (EMT) and activating AKT/mTOR signaling. Subsequently, ATP5A1 was determined as a potential target of HSPD1 using immunoprecipitation followed by mass spectrometry. Mechanistically, HSPD1 may interact with ATP5A1 to reduce the K48-linked ubiquitination and degradation of ATP5A1, which ultimately activates the AKT/mTOR pathway to ensure osteosarcoma progression and EMT process. These findings expand the potential mechanisms by which HSPD1 exerts biological effects and provide strong evidence for its inclusion as a potential therapeutic target in osteosarcoma.

## Introduction

Osteosarcoma, a primary malignant bone tumor, is known for its variability and aggressiveness, with frequent recurrences, high mortality rates, and a grim outlook. It is a major contributor to cancer-related fatalities in young individuals 1. For many years, neoadjuvant chemotherapy followed by surgery has been the mainstay of osteosarcoma treatment. While localized osteosarcoma typically responds well to treatment initially, the 5-year survival rate for patients with distant metastases decreases significantly to 20% 2. Regrettably, the early stages of osteosarcoma do not present with any distinct symptoms or signs, with around 15-20% of patients already having distant metastases upon initial diagnosis 3-6. The global burden of osteosarcoma is increasing significantly due to limited therapeutic options and a lack of characterized molecular targets 7, 8. This evidence suggests an urgent need to further explore clinically useful biomarkers to facilitate individualized treatment of osteosarcoma.

Non-oncogene addiction (NOA) reflects the hyperactivation of stress-management cellular pathways required for tumor cell survival, which not only confers an advantage to malignant cells by helping them grow but also creates a "cold" tumor microenvironment (TME) that aids in tumor advancement 9. Heat shock proteins (HSPs) represent examples of NOA that are critical for supporting the oncogenic phenotype of stressed tumor cells 10, 11. HSPs are a set of proteins that have been preserved throughout evolution and are increased in reaction to environmental or pathological stressors 12. They function as molecular chaperones to preserve the structure and activity of cellular proteins, as well as mediate de novo protein folding, maintenance of protein homeostasis, targeted transport, and assembly/disassembly of protein complexes 13, 14. HSPs not only prevent the aggregation of misfolded and denatured proteins but also cooperate with cellular degradation mechanisms to remove severely damaged proteins and abnormal protein aggregates 15. Throughout tumorigenesis, cancer cells exhibit increased vulnerability to proteotoxic stress as a result of various factors such as elevated metabolic activity, altered energy metabolism, and aneuploidy 16, 17. The persistent activation of the heat shock response pathway controlled by heat shock factor protein 1 is one of the recurrent features of malignant cells, which may reflect a cytoprotective response to the harsh conditions of the tumor microenvironment 18. This hijacking of physiological phenomena has also been described as “NOA” in cancer cells 19. Stress-induced HSPs in cancer cells are essential for the refolding and stabilization of many misfolded oncoproteins, allowing mutant proteins to be retained within the tumor and conferring resistance to cytotoxic treatments 20. Furthermore, it has been demonstrated that programmed cell death is one of the fundamental mechanisms of HSP-mediated modulation, including apoptosis, autophagy, necroptosis, and ferroptosis, among others 21. The HSP network is crucial in helping cancer cells avoid apoptosis. HSPs inhibit apoptosis through the suppression of the mitochondria-mediated endogenous apoptotic pathway, as well as through the modulation of other extrinsic death receptor signaling pathways 22. The frequent presence of high levels of HSPs in tumor tissues is expected, given their roles in controlling key biological functions in cancer cells such as proliferation, metabolism, differentiation, invasion, metastasis, and anti-apoptotic activity 23. Overexpression of HSP70 has been reported to confer cisplatin resistance to human ovarian cancer by preventing Bax translocation and inhibiting cisplatin-induced release of mitochondrial proteins 24. DNAJC12 reduced doxorubicin sensitivity in breast cancer cells by inhibiting ferroptosis and apoptosis through the activation of HSP70 and upregulation of AKT phosphorylation 25. HSP90AA1 inhibited osteosarcoma cell apoptosis by deactivating the JNK/P38 pathway and promoted autophagic protection in response to chemotherapy by blocking the PI3K/AKT/mTOR signaling pathway, thus serving as an important regulator of osteosarcoma survival 26. Remarkably, cancer cells exhibit higher metabolic demands and signaling activities to sustain their development and invasion compared to normal cells, which further increases the need for protein quality mechanisms 27. HSPs, particularly HSP90, HSP70, and HSPD1, also play pivotal roles in regulating cancer cell metabolism and metabolic reprogramming 28, 29.

HSPD1, also known as Chaperonin 60 or HSP60, is a member of the HSP family 30, 31. HSPD1 could act as a molecular chaperone in mitochondria, helping to import and fold proteins, and thereby participating in reprogramming cellular metabolism 23. HSPD1 deficiency triggers the mitochondrial unfolded protein response, leading to mitochondrial dysfunction and stemness loss 32. In addition, HSPD1 is involved in cellular signaling and may contribute to pro-survival or pro-apoptotic pathways, as appropriate. It may also be present on the cell surface or released extracellularly to interact with the immune system 30. Cytoplasmic HSPD1 appears to originate from mitochondria or to be synthesized de novo in the cytoplasm, whose origin and function are the subject of ongoing controversy 33. During carcinogenesis, the cellular distribution of HSPD1 undergoes a notable change, accumulating at extramitochondrial sites such as the cytoplasm, plasma membrane, and secretory vesicles 34. In the context of cancer, HSPD1 may play a different role, and interventions targeting HSPD1 are expected to destabilize invasive cancer cells and enhance antitumor immunity 34-36. Overexpression of this gene is commonly found in both non-solid and solid tumors, often leading to negative outcomes and the advancement of cancer 37, 38. HSPD1 exerts a wide range of anti-apoptotic functions in cancerous cells by mechanisms that include blocking p53, enhancing survivin stability, preventing mitochondrial permeability transition via cyclophilin D, and activating the IKK/NF-kappaB survival pathway 31, 39. As for the implication on metastatic phenotypes, HSPD1 collaborates with β-catenin to enhance metastasis by elevating the protein levels and transcriptional activity of β-catenin in head and neck cancer 36. HSPD1 oxidation activates the MAPK signaling pathway, which in turn increases migration-related genes and G1 cell cycle arrest in hepatocellular carcinoma (HCC) 40. Metabolically, HSPD1 maintains oxidative phosphorylation (OXPHOS) for the generation of adenosine triphosphate (ATP). Inhibition of HSPD1 induces apoptosis and cell cycle arrest by decreasing Erk1/2 phosphorylation and reducing endogenous mitochondrial ATP production in pancreatic ductal adenocarcinoma 41. Additionally, HSPD1 is actively secreted by malignant cells and participates in processes such as transformation, angiogenesis, and metastasis 42, 43. Conversely, HSPD1 may also be associated with tumor suppressors. As an illustration, HSPD1 is crucial for the correct folding and mitochondrial input of Fhit 44. Overexpression of Fhit proteins regulates intracellular reactive oxygen species production, induces apoptosis, and inhibits tumorigenicity under conditions of oxidative stress 44. As a tumor suppressor, HSPD1 induces apoptosis by accelerating the maturation of caspase-3 precursors through upstream activation of proteases 45. Additionally, it has been demonstrated that HSPD1 inhibits ovarian cancer proliferation and migration by stabilizing mitochondrial protein and lipoic acid synthesis 46. The diverse functions of HSPD1 in cancer may stem from the activation of various molecular pathways in different types of cancer, highlighting the intricate and diverse nature that defines the complexity of cancer biology. The diverse array of gene mutations, gene expression patterns, and signaling pathways observed in different cancer types may contribute to the varying roles of HSPD1 in different cancers. Nevertheless, the relationship between the function of HSPD1 and the progression of osteosarcoma remains unclear. Consequently, further investigations are required to elucidate the precise function of HSPD1 in osteosarcoma, which will facilitate the comprehension of its specific mechanistic role across different cancer types.

In this study, we analyzed bulk and single-cell transcriptomic data from publicly available osteosarcoma cohorts (GEO and TARGET datasets) to identify HSPs associated with adverse patient prognosis. Our study revealed two distinct HSP modification patterns and noted contrasting biological functions between them. Considering the progression and intratumoral heterogeneity of osteosarcoma, we established the HSP scoring system to quantify the risk stratification of individual patients and validated its potential application in TME, clinicopathological characterization, and prognosis. Based on multi-omics analysis and preliminary experiments, we determined that aberrant overexpression of HSPD1 is strongly associated with impaired outcomes in osteosarcoma and acts as a tumor promoter. Our study details the biological role of HSPD1 *in vitro* and *in vivo*, the corresponding functional mechanisms, and its potential relationship with ATP5A1 to identify possible treatment options for osteosarcoma.

## Methods

### Cell lines

The human osteoblast cell line (hFOB1.19) and the osteosarcoma cell lines (MG63 and MNNG/HOS) were obtained from the China Center for Type Culture Collection in Shanghai, China. U2OS osteosarcoma cell line and HEK-293T cell line were acquired from Pricella Biotechnology Co., Ltd in Wuhan, China. The HEK-293T cell line and all osteosarcoma cell lines were grown in the appropriate medium with 10% fetal bovine serum and 100 U/ml penicillin/streptomycin at 37°C and 5% CO2, except for hFOB1.19 cells, which were kept in a 5% CO2 environment at 33.5°C. These cells were authenticated by STR identification prior to freezing.

### Quantitative real-time reverse transcription-PCR (qRT-PCR)

RNA was isolated using RNA-easyTM reagent from Vazyme Biotech Co., Ltd., Nanjing, China, followed by cDNA synthesis using HiScript III RT SuperMix from Vazyme. The SYBR Green PCR Master Mix from CWBIO was utilized for qRT-PCR analysis with the Bio-Rad CFX96 instrument (CFX96, Bio-Rad, USA).The 2-ΔΔCT method was used to calculate the relative gene expression with β-actin as the internal control. The** supplementary Table S1** contains information on primer sequences for pertinent genes.

### Plasmid construction and transfection

IGE BIO (Guangzhou, China) created plasmids for the overexpression of HSPD1, plasmids for the overexpression of ATP5A1, and empty vectors. RiboBio (Guangzhou, China) synthesized shRNA sequences targeting HSPD1 and ATP5A1 for gene downregulation, which are detailed in **supplementary Table S2**. Lipofectamine 3000 (Invitrogen, USA) was utilized for transfection in accordance with the provided guidelines. Transfection efficiency was measured 24-48 hours after transfection by qRT-PCR or western blotting.

### Cell Counting Kit-8 (CCK-8) assay and colony-forming assay

Cell viability was assessed by a colony formation assay in which 1×10^3^ osteosarcoma cells were inoculated in 6-well plates and cultured for 12 days before staining with crystal violet. In the CCK-8 test, cells were placed in a 96-well plate at a concentration of 1×10^3^ cells per well. At specific time intervals, 10 microliters of CCK-8 solution were introduced. Following one hour of incubation at 37°C, the optical density was assessed at 450 nm using a microplate reader.

### Wound healing and transwell assay

Cell migration and invasion abilities were analyzed through the performance of Transwell and wound healing assays. Additional information on the procedures can be found in the **supplementary materials and methods**.

### Western blotting (WB) and coimmunoprecipitation (Co-IP)

Proteins were isolated using RIPA lysis buffer from Beyotime in China, which included protease and phosphatase inhibitors. The lysates were then gathered and centrifuged at 4°C (12,000 rpm, 15 min). Following separation using SDS-PAGE gels from Epizyme in Shanghai, China, the proteins were moved to PVDF membranes from Millipore in the USA and then exposed to the appropriate primary antibodies overnight at 4 °C. Following TBST wash, the membranes were exposed to secondary antibodies labeled with HRP for 1 hour at ambient temperature and visualized with a chemiluminescent kit from Epizyme in Shanghai, China. Details of the antibodies utilized are described in **supplementary materials and methods**.

For the CO-IP assay, samples were obtained using IP buffer (Beyotime, China). After determination of protein concentration with a protein assay kit (Thermo Fisher, USA), supernatant fractions containing equal amounts of protein were immunoprecipitated with the corresponding antibodies, with a non-specific immunogen IgG antibody (normal mouse IgG, sc2025, Santa Cruz, USA; normal rabbit IgG, 2729S, CST, USA) as a negative control. The immunoprecipitated complexes were obtained using protein A/G plus agarose beads (SC2003, Santa Cruz, USA) and eluted for WB analysis.

### LC-MS/MS analysis for quantitative proteomics

Sample preparation and proteomic analyses were performed as described previously 47. Additional information can be found in **supplementary materials and methods**.

### *In vivo* xenograft assay

The Animal Ethics Committee of Jinan University approved all animal experiments (Approval No. IACUC-20231213-07) which were carried out at the Animal Experiment Centre of Jinan University. The BALB/c immunodeficient mice used in this experiment were obtained from Charles River (Charles River, China) and then grouped randomly into four groups, each containing 6 mice. Subcutaneous and orthotopic osteosarcoma models were created by implanting control or stable HSPD1 silenced MNNG/HOS cells (4×10^6^ cells in 0.1 ml PBS) into nude mice. Every 5 days, the mice's tumor volume and growth status were documented. Tumor volume was calculated as follows: volume (mm^3^) = (width)^2^ × length × 0.52. After 25 days of observation, all mice were executed to isolate tumor specimens for analysis.

### Flow cytometry

For apoptosis rate assay, transfected osteosarcoma cells were digested with EDTA-free trypsin (NCM Biotech, Suzhou, China). Cell culture medium and adherent cells were collected and double stained with Annexin V-FITC / PI apoptosis detection kit (556547, BD Pharmingen, USA). The apoptosis rate of osteosarcoma cells was detected and analyzed using a Gallios flow cytometer and Kaluza software (Beckman Coulter, USA).

Treated osteosarcoma cells were harvested, washed with PBS, and fixed in ice-cold 70% ethanol at 4°C overnight for cell cycle analysis. After two washes with cold PBS, fixed cells were incubated with 500 μL PI/RNase staining buffer (550825, BD Pharmingen, USA) at room temperature. Each sample was analyzed using a Gallios flow cytometer and Multicycle software (Beckman Coulter, USA).

### Protein stability assay and ubiquitination assay

The stability of ATP5A1 protein in osteosarcoma cells was determined using a pulse-chase assay with cycloheximide (CHX, 100 μM, MedChemExpress, Shanghai, China). Cells were harvested and lysed at different time points to determine the half-life of the ATP5A1 protein. For ubiquitination assays, cells were pre-treated with the proteasome inhibitor MG132 (10 μM, MedChemExpress, Shanghai, China) for 6 h. Cell lysates obtained after transfection were incubated with anti-HA antibody or anti-ATP5A1 antibody (14676-1-AP, Proteintech, China) at 4°C overnight, followed by incubation with protein A/G plus agarose beads at 25°C for 4 hours. After the beads were thoroughly washed, the level of ATP5A1 ubiquitination was assessed by WB.

### Haematoxylin-eosin (HE) and immunohistochemistry (IHC) staining

IHC and HE staining were conducted as described previously 48. Additional information is available in **supplementary materials and methods**.

### Bioinformatics data collection and analysis

We screened the osteosarcoma cohorts in two publicly available databases, GEO (https://www.ncbi.nlm.nih.gov/geo/) and TARGET (https://ocg.cancer.gov/programs/target). In the TARGET cohort, 85 osteosarcoma patients with clinically documented and corresponding bulk RNA-seq data were obtained. In the GSE21257 cohort, 53 osteosarcoma patients with complete follow-up data and sequencing data were included in the survival analysis. The bulk RNA-seq data was normalized into transcripts per kilobase million format and log2-transformed. In cases of duplicate data, the mean RNA expression level was utilized. The gene expression matrices mentioned above were merged to create an osteosarcoma meta-cohort consisting of 138 samples, and any bias was corrected using the 'SVA' package. As a control, we acquired 396 typical musculoskeletal samples from the GTEx database (https://www.gtexportal.org/home/). Transcriptome expression datasets of cancer cell lines were downloaded from CCLE (https://sites.broadinstitute.org/ccle). The expression of HSPs at the single-cell level was analyzed using single-cell RNA sequencing (scRNA-seq) data from six osteosarcoma samples in the GSE162454 dataset. Data regarding HSPD1 expression patterns and related clinical data for pan-cancer samples were acquired from The Cancer Genome Atlas (TCGA) database. The gene list of HSPs was generated from the published literature **(supplementary Table S3)** 37, 49-54. The overall bioinformatics analysis strategy of this study was as follows: expression pattern and prognostic significance of HSPs in osteosarcoma; evaluation of the potential biological functions of diverse HSP molecular subtypes; development and validation of the HSP-based risk stratification system for predicting the clinical prognosis, tumor immune microenvironment, drug sensitivity, and immunotherapy efficacy; Single-cell analysis of HSPD1. Analytical methods used include consensus clustering analysis, differential expression analysis, LASSO regression, gene set enrichment analysis (GSEA), gene set variation analysis (GSVA), single-sample GSEA (ssGSEA) analysis, subcellular localization analysis, and COX regression. Detailed descriptions of these bioinformatic analyses are available in the **supplementary materials and methods**.

### Statistical analysis

Statistical analysis was conducted using R version 4.3.0 and GraphPad Prism 8 software. Each experiment was conducted with a minimum of three biological replicates. Group variances were compared using unpaired two-tailed Student's t-tests and one-way ANOVA. P values < 0.05 were considered statistically significant, and error bars indicate calculated SD values.

## Results

### Prognostic significance and molecular subtypes of HSPs in osteosarcoma

**Figure 1:**
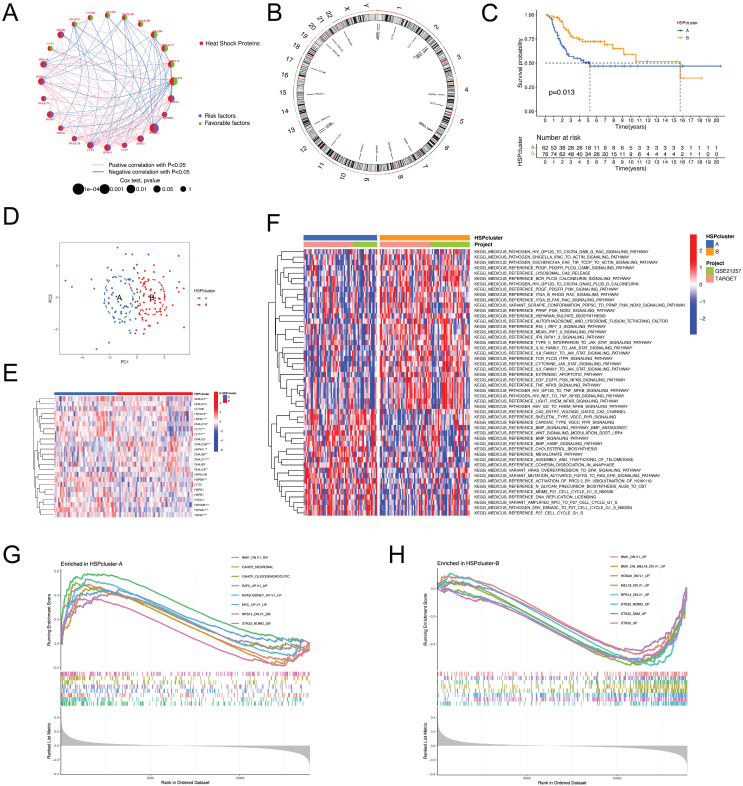
** Determination of HSP molecular subtypes and characterization of biological traits for each pattern. (A)** Interactions between HSPs in osteosarcoma, with circle size representing the prognostic impact of each HSP.** (B)** Location of prognostic HSPs on the chromosome. **(C)** Survival analyses for the two distinct HSP patterns showed a significant survival difference. **(D)** Principal component analysis for the transcriptome profiles of two HSP patterns in osteosarcoma. **(E)** The expression of 24 prognostic HSPs between two distinct phenotypes.** (F)** GSVA enrichment analysis reveals distinct activation states of biological pathways in HSP molecular subtypes.** (G-H)** GSEA enrichment analysis of HSPcluster A and B using C6 oncogenic signature gene sets.

We analyzed the association of HSPs with prognosis by combining the TARGET and GEO cohorts, and univariate Cox regression analysis revealed that 24 HSPs exerted a remarkable impact on the outcome in patients with osteosarcoma** (Figure S1A)**. The 14 adverse prognostic HSPs were largely positively correlated with each other but showed significant negative correlations with 10 favorable prognostic HSPs **(Figure 1A)**. The chromosomal location of these prognostic HSPs is shown in **Figure 1B**. Patients were then categorized by unsupervised clustering to investigate the impact of HSPs on the formation of various molecular subtypes. Ultimately, two distinct HSP patterns, subsequently named HSPcluster A-B, were identified (62 cases in HSPcluster-A and 76 cases in HSPcluster-B), with the HSPcluster-B showing a significant survival advantage **(Figure S1B-D, Figure 1C)**. PCA analysis further demonstrated superior grouping** (Figure 1D)**. Furthermore, we investigated the transcriptional profiles of HSPs between two distinct phenotypes and found that HSPs considered favorable indices (including HSPA1L, DNAJB5, DNAJB7, DNAJC5B, DNAJC8, and DNAJC17) exhibited significantly higher expression in HSPcluster-B **(Figure 1E)**. Conversely, adverse prognostic HSPs (e.g., HSPA1A, HSPA4L, HSPB1, HSPB7, and HSPB8) showed significantly higher expression in HSPcluster-A **(Figure 1E)**.

### Distinct functional annotations in the two HSP phenotypes

GSVA enrichment analysis was conducted to determine if the prognostic variances are associated with the biological characteristics and activities of the two separate HSP molecular subtypes. **Figure 1F** demonstrates that HSPcluster-A exhibited enrichment in biosynthesis, cell cycle, and cancer-related pathways, including cholesterol biosynthesis, amplified MYC to P27 cell cycle G1-S, and HRAS overexpression to ERK signaling pathway. The most significantly enriched pathways of HSPcluster-B are associated with cancer and inflammatory responses, including type II interferon to JAK-STAT signaling pathway, IL10 family to JAK-STAT signaling pathway, EGF EGFR PI3K NFKB signaling pathway, and TNF NFKB signaling pathway **(Figure 1F)**. GSEA analysis using MSigDB (C6 oncogenic signature gene sets) was used to further explore the potential association between HSP modification patterns and oncogenic pathways. The gene sets representing the STK33, MEL18, and BMI1 transcriptional programs were particularly enriched in HSPcluster-B, while HSPcluster-A showed enrichment of the oncogenic signature gene set upregulated by MYC expression, E2F3 expression, and KRAS expression **(Figure 1G-H)**.

### Development of the HSP-derived scoring system and related clinical application

**Figure 2:**
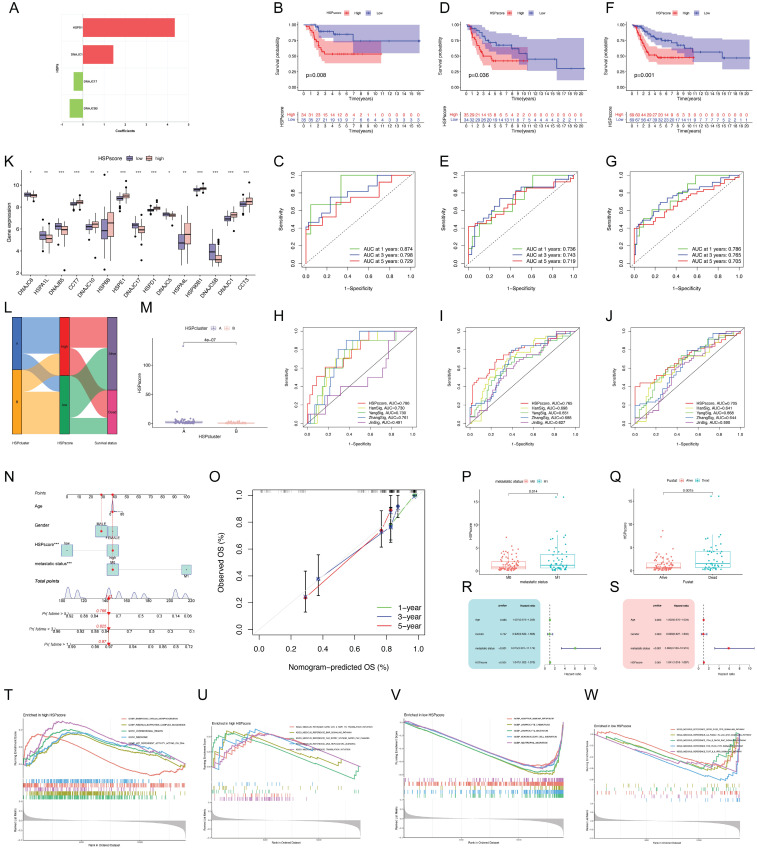
** Generation of the HSP-derived scoring system and associated clinical applications. (A)** Coefficients for four prognostic HSPs in the HSP-derived scoring system using the multivariate Cox analysis. **(B)** Survival analysis for different HSPscore groups in training cohort using Kaplan-Meier curves. **(C)** ROC curves for 1-, 3-, and 5-year survival in the training cohort. **(D)** Survival analysis for different HSPscore groups in validation cohort using Kaplan-Meier curves. **(E)** ROC curves for 1-, 3-, and 5-year survival in the validation cohort. **(F)** Survival analysis for different HSPscore groups in the entire cohort using Kaplan-Meier curves. **(G)** ROC curves for 1-, 3-, and 5-year survival in the entire cohort. **(H-J)** Comparison of HSP-derived scoring system with published prognostic signatures using time-dependent ROC curve analysis of 1-year **(H)**, 3-year **(I)**, and 5-year **(J)** overall survival predictions. **(K)** The expression of prognostic HSPs between different HSPscore groups. **(L)** Alluvial diagram showing the changes of HSP molecular subtypes and HSPscore. **(M)** Differences in HSPscore between HSPcluster A and B. **(N)** Nomogram based on the HSPscore and clinical parameters.** (O)** Nomogram calibration curves for 1-, 3-, and 5-year overall survival.** (P-Q)** Differences in HSPscore among patients with different metastatic status** (P)** and survival status **(Q)**. **(R-S)** Univariate** (R)** and multivariate **(S)** Cox regression analyses to identify the prognostic significance of HSPscore and clinical parameters. **(T-U)** Gene Ontology** (T)** and Kyoto Encyclopedia of Genes and Genomes** (U)** enrichment in the high HSPscore group by GSEA analysis. **(V-W)** Gene Ontology **(V)** and Kyoto Encyclopedia of Genes and Genomes **(W)** enrichment in the low HSPscore group by GSEA analysis.

The above analyses demonstrate the non-negligible correlation between HSP molecular subtypes and the prognosis of osteosarcoma patients but only based on patient populations. We hypothesized that certain markers reflecting HSP phenotypes could be used to construct prognostic models predicting patterns of HSP modifications in individual patients. Four center prognostic HSPs were identified using LASSO and multivariate COX regression analyses to construct the HSP-derived scoring system **(Figure S2A-C)**. **Figure 2A** illustrates the coefficients for each HSP in the scoring system. HSPscore = (HSPD1 * 4.343358) + (DNAJC1 * 1.435962) - (DNAJC5B * 0.629422) - (DNAJC17 * 0.440761). The training cohort was split into two groups based on median HSPscore, revealing that the high-HSPscore group of osteosarcoma patients had notably poorer overall survival (OS) with AUCs of 0.874, 0.798, and 0.729 at 1, 3, and 5 years **(Figure S2D, Figure 2B-C)**. In order to confirm its applicability, the scoring system derived from HSP was applied to two additional combined bulk RNA-seq cohorts, showing once again that patients with low HSPscore had a notable survival advantage with AUC exceeding 0.7 at 1, 3, and 5 years **(Figure S2E-F, Figure 2D-G)**. We sought to further determine the value of HSPscore in predicting patient outcomes and therefore compared the known signature of osteosarcoma with HSPscore by ROC analysis, further confirming the quantification of HSPscore as a potential and reliable prognostic biomarker **(Figure 2H-J)**. A notable difference was observed in the HSP transcriptional profile between individuals with high and low HSP scores** (Figure 2K)**. The high HSPscore tumors were characterized by the increased expression of CCT7, DNAJC10, HSPB8, HSPE1, HSPD1, HSPA4L, HSP90B1, DNAJC1, and CCT3; and osteosarcoma patients with depressed HSPscores exhibited significant increases in the expression of DNAJC8, HSPA1L, DNAJB5, DNAJC17, DNAJC5B, and DNAJC5 **(Figure 2K)**.

The alluvial diagram illustrates the variation in attributes of individual patients, with the majority of cases in HSPcluster-B assigned to the low-HSPscore group and most patients in HSPcluster-A assigned to the high-HSPscore group **(Figure 2L)**. More importantly, HSPcluster-A exhibited significantly increased HSPscores **(Figure 2M)**. We specifically integrated HSPscore and important clinical factors (including patient age, gender, and metastatic state) to construct nomograms that can accurately predict the survival time of individuals with osteosarcoma **(Figure 2N)**. There was a good consistency between the predicted probabilities and the actual observed outcomes as shown by the calibration curves **(Figure 2O)**. To better illustrate the characteristics of the HSP-derived stratification framework, we tested the correlation between the aforementioned clinicopathologic features and the HSPscore. Osteosarcoma patients without metastasis showed lower HSPscore compared to those with metastasis **(Figure 2P)**. HSPscore also differed significantly between patients with different survival statuses, with higher HSPscore in deceased patients **(Figure 2Q)**. Cox regression analyses verified that the HSPscore served as an independent prognostic biomarker in evaluating osteosarcoma outcomes** (Figure 2R-S)**.

To further investigate the underlying biological mechanisms in patients with different HSPscores, we performed GSEA analyses. DNA replication or translation-related cellular functions and pathways were significantly enriched in high HSPscore groups, such as ribonucleoprotein complex biogenesis, translation initiation, and DNA replication licensing **(Figure 2T-U);** whereas adaptive immune response, lymphocyte chemotaxis, lymphocyte migration, mononuclear cell migration, neutrophil migration, and TCR-PLCG-ITPR signaling pathway were considerably increased among low HSPscore patients** (Figure 2V-W)**. We hypothesize that the low-HSPscore subgroups in an immune-activated state may be closely related to the apparent upregulation of biological processes associated with innate immune responses and adaptive immunity.

### Immune landscape analysis and precision treatment of osteosarcoma following the HSP-derived scoring system

**Figure 3:**
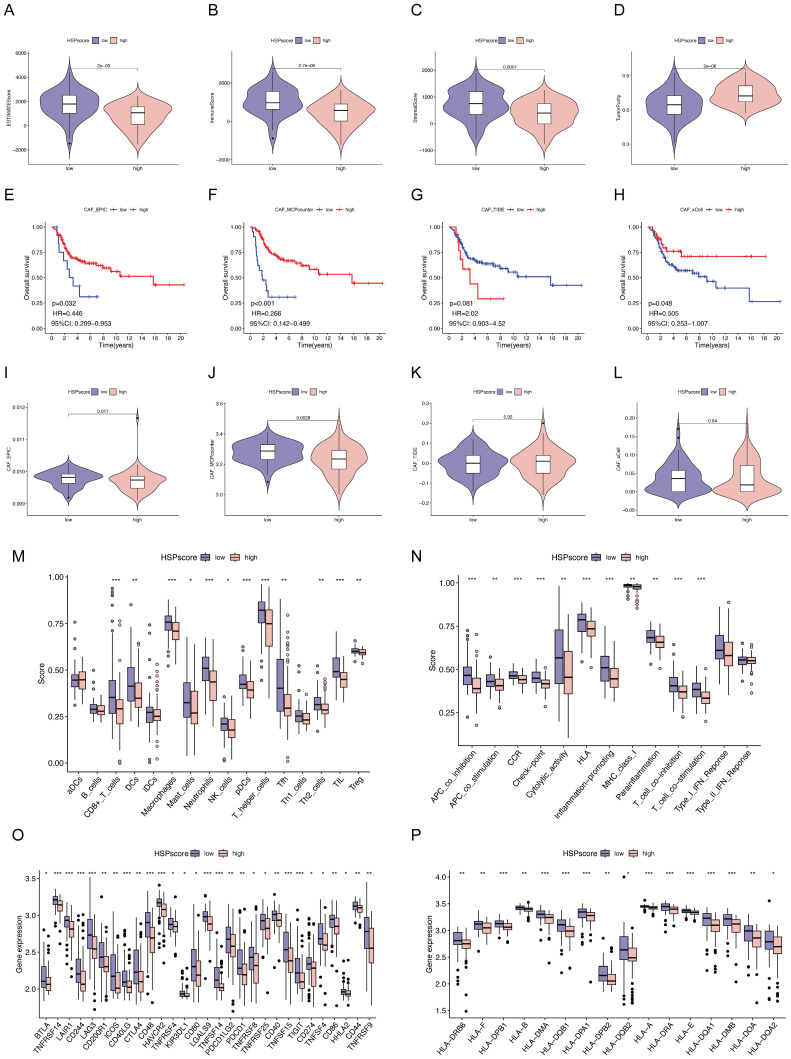
** Immune landscape analysis, immunotherapy response prediction, and drug susceptibility analysis following the HSPscore. (A-D)** Differences of ESTIMATEScore **(A)**, ImmuneScore **(B)**, StromalScore **(C)**, and tumor purity **(D)** between low and high HSPscore groups.** (E-H)** Kaplan-Meier analysis for overall survival based on CAF proportion scores of osteosarcoma patients derived by EPIC **(E)**, MCP-Counter **(F)**, TIDE **(G)**, and xCell **(H)** algorithms.** (I-L)** Differences in CAF proportion scores between low and high HSPscore groups were calculated by EPIC **(I)**, MCP-Counter **(J)**, TIDE** (K)**, and xCell** (L)** algorithms. **(M-N)** Differential analysis of tumor-infiltrating immune cells **(M)** and immune functions **(N)** was performed using the ssGSEA algorithm. **(O)** Differences of immune checkpoint gene expression between high and low HSPscore groups.** (P)** Differential MHC molecules expression between high and low HSPscore groups.

Subsequently, we attempted to characterize the immune landscape according to the HSP-derived scoring system, given the huge impact of TME on malignant neoplasm progression and immunotherapy. The ESTIMATE algorithm was used to quantify the overall immune cell infiltration between high and low HSPscore groups. A notable rise in tumor purity and a decrease in ESTIMATE score, immune score, and stromal score were noted as HSPscore increased from low to high levels** (Figure 3A-D)**. Cancer-associated fibroblasts (CAFs), an important stromal component of the TME with extensive crosstalk with infiltrating immune cells, have long been considered an attractive therapeutic target 55. Using the MCPCounter, xCELL, EPIC, and TIDE algorithms, we calculated the proportion of CAFs for each osteosarcoma sample and observed that the lower the proportion of CAFs, the poorer the survival outcomes in the three algorithms **(Figure 3E-H)**. There was a strong correlation and variability between HSPscore and the abundance of CAFs. Specifically, the abundance of CAFs was lower in the high HSPscore group by the EPIC and MCPCounter algorithms, and conversely, there was no notable difference in CAF abundance between the low and high HSPscore groups by the xCELL and TIDE algorithms **(Figure 3I-L)**.

Specific variations in TME-infiltrating immune cells and immunological functions were also analyzed in different HSPscore categories using the ssGSEA algorithm. The group with low HSPscore had a remarkable presence of both innate and adaptive immune cells, including CD8(+) T cells, dendritic cells (DCs), macrophages, mast cells, neutrophils, plasmacytoid dendritic cells (pDCs), tumor-infiltrating lymphocytes (TILs), natural killer (NK) cells, T helper cells, T follicular helper (Tfh) cells, Th2 cells, and regulatory T cells (Tregs) **(Figure 3M, Figure S3)**. Meanwhile, in the high HSPscore group, the pathways of APC co-inhibition, APC co-stimulation, cytokine-cytokine receptor (CCR), checkpoint, human leukocyte antigen (HLA), cytolytic activity, inflammation-promoting, MHC class I, parainflammation, T cell co-inhibition, and co-stimulation pathways were notably inhibited **(Figure 3N, Figure S3)**. Concurrently, we analyzed the levels of immune checkpoint genes (ICGs) and major histocompatibility complex (MHC) molecules in individuals with varying HSPscore. Low HSPscore groups exhibited a greater abundance of ICGs and MHC molecules **(Figure 3O-P)**. Consistent with the above findings, HSPscore was negatively correlated with the expression of immune checkpoint and MHC molecules by Spearman correlation analysis **(Figure S4-5)**.

Maintenance therapy and chemotherapy after tumor-reducing surgery in osteosarcoma patients are crucial 56. The sensitivity of individuals in different HSPscore groups to common antineoplastic drugs was further evaluated using the Genomics of Drug Sensitivity in Cancer database. Patients with low HSPscore were more sensitive to Bcl-2/Bcl-xL inhibitors (ABT-737), PLK1 inhibitors (BI-2536), and AURORA kinase inhibitors (tozasertib), but less sensitive to CDK4/6 inhibitor (ribociclib), JAK1/2 inhibitor (ruxolitinib), and MEK1/2 inhibitor (selumetinib) **(Figure S6)**. In conclusion, these results suggest that the HSPscore provides valuable insights into the immune status and underlying tumor biology of individual osteosarcoma patients and is a valuable tool to guide therapeutic decisions and improve prognosis.

### HSPD1 is highly expressed in osteosarcoma cells as a potential prognostic marker by multi-omics analysis

**Figure 4:**
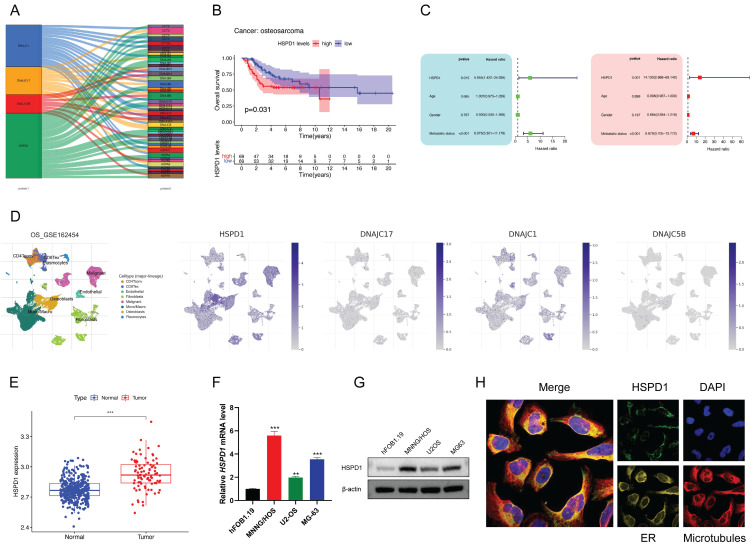
** Multi-omics analysis identifies HSPD1 as highly expressed in osteosarcoma and a potential prognostic marker. (A)** Alluvial diagram showing the correlation between core HSPs and prognostic HSPs.** (B)** Kaplan-Meier survival analysis of osteosarcoma patients stratified by HSPD1 expression level.** (C)** Univariate and multivariate COX analyses to identify the prognostic significance of HSPD1 and pathological indicators in osteosarcoma. **(D)** Single-cell RNA sequencing analysis of HSPD1 expression in malignant osteosarcoma cells and immune cell subpopulations. UMAP plots for visualizing the abundance distribution of four core HSPs (HSPD1, DNAJC1, DNAJC5B, and DNAJC17), with color gradients showing the normalized expression levels. **(E)** Bulk RNA sequencing analysis of HSPD1 expression in osteosarcoma and normal tissues.** (F-G)** Expression of HSPD1 in hFOB1.19 cells and osteosarcoma cell lines detected by qRT‒PCR** (F)** and Western blot** (G)**.** (H)** Immunofluorescence images depicting subcellular localization of HSPD1 in U2OS cells.

The inter-regulatory connections between the four core HSPs and prognostic HSPs expressed in osteosarcoma are visualized in Sankey diagrams **(Figure 4A)**. More importantly, HSPD1 showed broader associations with prognostic HSPs compared to the other three core HSPs. Although clinical outcomes were generally better in patients with higher DNAJC5B and DNAJC17 expression, the difference was not statistically significant **(Figure S7A-B)**. In contrast, elevated HSPD1 and DNAJC1 expression were associated with inferior outcomes in osteosarcoma **(Figure 4B, Figure S7C)**. Considering that HSPD1 not only had the highest hazard ratios in univariate Cox regression but also the highest positive coefficients in the HSP-derived scoring system, we next focused on analyzing the multi-omics data of HSPD1 in osteosarcoma. Univariate and multivariate analyses further indicated that HSPD1 was a significant prognostic factor for osteosarcoma independent of other clinical variables, as depicted in **Figure 4C (HR, 14.130; 95%CI, 2.888-69.140)**.

Within the scRNA-seq information for osteosarcoma (GSE162454), HSPD1 was detected in both cancerous cells and non-cancerous cells like endothelial cells, CD4+ T conventional lymphocytes, macrophages, and plasmocytes** (Figure 4D)**. Notably, HSPD1 was expressed at the highest abundance in malignant cells compared to other core HSPs** (Figure 4D)**. Bulk RNA-seq data indicated a notable increase in HSPD1 expression in osteosarcoma tissues compared to normal tissues** (Figure 4E)**. Comparing HSPD1 expression in normal osteoblasts (hFOB1.19) against each of the three different osteosarcoma cells (U2OS, MG63, and MNNG/HOS) revealed significant upregulation of the gene in tumor **(Figure 4F-G)**. Subcellular localization analysis indicated predominant expression of HSPD1 in the mitochondria with immunofluorescence **(Figure 4H)**.

### HSPD1 knockdown inhibits osteosarcoma progression and metastasis

**Figure 5:**
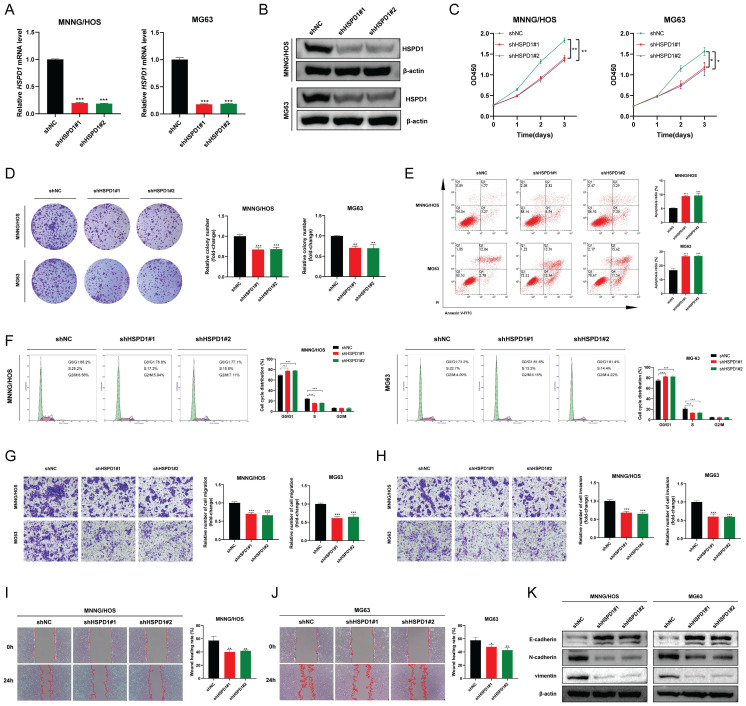
** HSPD1 silencing impairs osteosarcoma proliferation, metastasis, and epithelial-mesenchymal transition. (A)** Validation of HSPD1 downregulation in osteosarcoma cells by qRT-PCR analyses. **(B)** The efficacy of HSPD1 knockdown in MNNG/HOS and MG63 cells was verified by western blot.** (C)** Proliferative capacity of MNNG/HOS and MG63 cells with HSPD1 downregulation assessed by CCK-8 assay. **(D)** Colony formation assays assessing the clonogenic potential of osteosarcoma cells following HSPD1 silencing. **(E)** Flow cytometry analysis of apoptotic rate in HSPD1-knockdown osteosarcoma cells using the Annexin V-FITC/PI staining.** (F)** Flow cytometry analysis of cell cycle phase distribution in HSPD1-knockdown osteosarcoma cells. **(G)** Exploration of the migration capabilities of MNNG/HOS and MG63 cells with HSPD1 downregulation by transwell experiments.** (H)** Exploration of the invasion capabilities of MNNG/HOS and MG63 cells following HSPD1 silencing by transwell experiments. **(I-J)** Exploration of the migration capabilities of MNNG/HOS** (I)** and MG63** (J)** cells with HSPD1 downregulation by wound healing assay. **(K)** Western blot detected the expression of EMT markers in MNNG/HOS and MG63 cells treated with HSPD1 knockdown. The data are presented as mean ± SD. *P < 0.05; **P < 0.01; ***P < 0.001.

Establishing osteosarcoma cell lines with stable HSPD1 knockdown was done to determine if HSPD1 could influence malignant behavior, and the effectiveness of the knockdown was confirmed** (Figure 5A-B)**. CCK-8 assay and colony formation assay suggested that HSPD1 knockdown weakened the proliferation and colony-forming ability of MNNG/HOS and MG63 cells** (Figure 5C-D)**. Next, as shown in **Figure 5E**, HSPD1 downregulation increased the rate of cell apoptosis via Annexin-V-FITC staining. Meanwhile, flow cytometry experiments revealed that HSPD1 knockdown induced cell cycle arrest mainly in the G0/G1 phase, along with a decline in the S phase of osteosarcoma cells, compared with controls **(Figure 5F)**. Furthermore, transwell and wound-healing assays indicated that suppressing HSPD1 led to a reduction in the migration and invasion efficiency of MNNG/HOS and MG63 cells **(Figure 5G-J)**. Considering that EMT is critical for tumor invasion, migration, and metastasis, we examined the expression of EMT markers to investigate the mechanisms underlying a potential HSPD1-driven phenotype. As confirmed by Western blotting, the knockdown of HSPD1 resulted in the upregulation of epithelial marker E-cadherin, while a simultaneous decrease in mesenchyme markers vimentin and N-cadherin **(Figure 5K)**. Taken together, these data suggest that HSPD1 knockdown significantly inhibited the malignant biological behavior of osteosarcoma and enhanced apoptosis.

### HSPD1 overexpression induces EMT and progression of osteosarcoma

**Figure 6:**
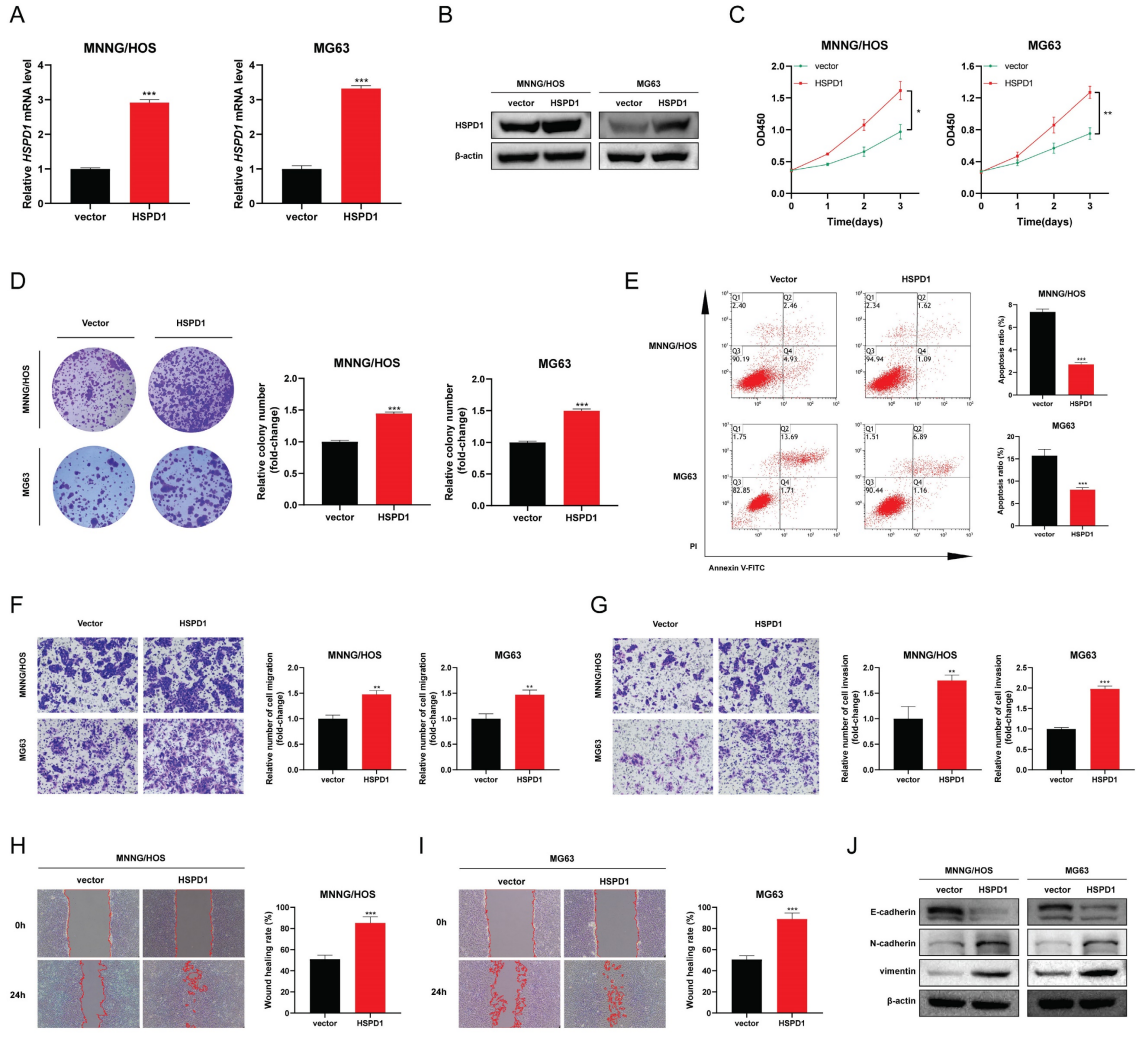
** Overexpression of HSPD1 exerts pro-cancerous activity and accelerates epithelial-mesenchymal transition in osteosarcoma. (A)** Validation of HSPD1 overexpression in osteosarcoma cells by qRT-PCR analyses.** (B)** The efficacy of HSPD1 overexpression in MNNG/HOS and MG63 cells was verified by Western blot. **(C)** Proliferative capacity of MNNG/HOS and MG63 cells with HSPD1 overexpression assessed by CCK-8 assay.** (D)** Colony formation assays assessing the clonogenic potential of osteosarcoma cells following HSPD1 upregulation.** (E)** Flow cytometry analysis of apoptotic rate in osteosarcoma cells stably overexpressing HSPD1 using Annexin V-FITC/PI staining. **(F)** Exploration of the migration capabilities of MNNG/HOS and MG63 cells with HSPD1 overexpression by transwell experiments. **(G)** Exploration of the invasion capabilities of MNNG/HOS and MG63 cells following HSPD1 overexpression by transwell experiments.** (H-I)** Exploration of the migration capabilities of MNNG/HOS** (H)** and MG63** (I)** cells with HSPD1 upregulation by wound healing assay.** (J)** Western blot detected the expression of EMT markers in MNNG/HOS and MG63 cells treated with HSPD1 upregulation. The data are presented as mean ± SD. *P < 0.05; **P < 0.01; ***P < 0.001.

HSPD1 was significantly augmented in MNNG/HOS and MG63 cells following transfection with HSPD1-overexpression plasmid **(Figure 6A-B)**. HSPD1 upregulation in osteosarcoma cells significantly promoted cell viability and increased the number and size of colony formation compared with vector controls **(Figure 6C-D)**. In contrast, the proportion of apoptosis was significantly reduced in HSPD1 overexpressing osteosarcoma cells **(Figure 6E).** In transwell assays, HSPD1 overexpression resulted in augmented migration and invasion efficiency of MNNG/HOS and MG63 cells **(Figure 6F-G)**. Consistently, wound scratch assay also showed that overexpression of HSPD1 accelerated wound healing **(Figure 6H-I)**. The results of WB analysis showed that overexpressed HSPD1 suppressed the expression of E-cadherin, and enhanced the expression of N-cadherin and vimentin in osteosarcoma cells **(Figure 6J)**. These changes further validate our results in the HSPD1 knockdown experiments and illustrate that HSPD1 accelerates tumorigenesis and EMT induction in osteosarcoma.

### HSPD1 enhances the AKT/mTOR signaling in osteosarcoma

**Figure 7:**
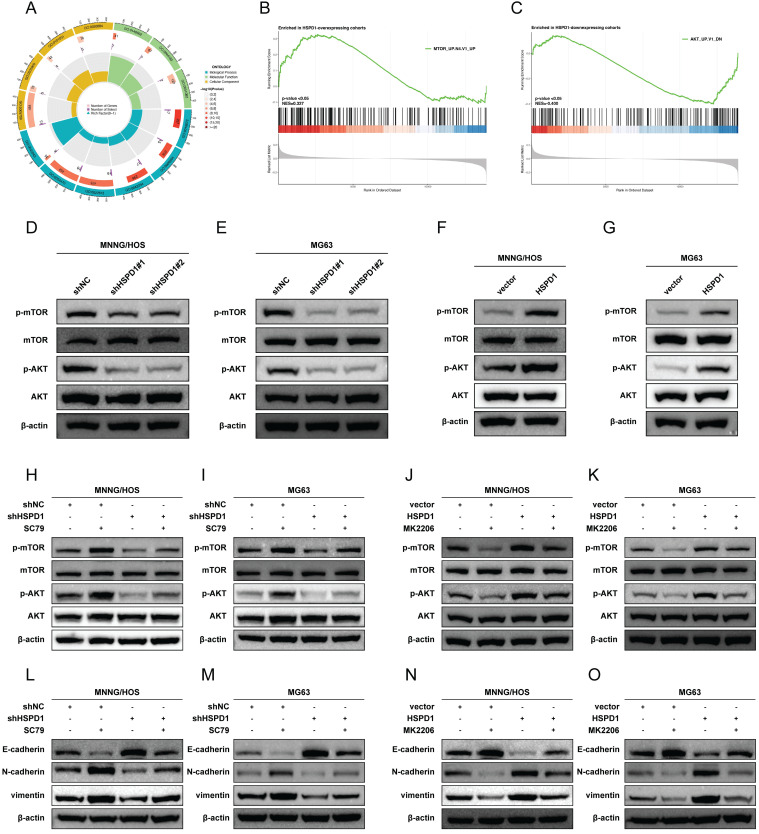
** HSPD1 enhances the AKT/mTOR signaling in osteosarcoma. (A)** Gene Ontology enrichment analysis of differentially expressed genes between high and low HSPD1 expression groups. **(B)** Gene set enrichment analysis revealed the up-regulation of mTOR signaling (C6 oncogenic signature gene set: MTOR_UP.N4.V1_UP) in HSPD1-overexpressing cohorts. The median value of HSPD1 transcriptional level was regarded as the cut-off to divide osteosarcoma patients into high- or low-HSPD1 expression groups.** (C)** Gene set enrichment analysis revealed the AKT-downregulated gene signature (AKT_UP.V1_DN ) enriched in the low HSPD1 expression groups.** (D-E)** Western blot assay revealed the expression of AKT, p-AKT, mTOR, and p-mTOR in MNNG/HOS** (D)** and MG63 **(E)** cells after stable HSPD1 silencing.** (F-G)** Western blot assay revealed the expression of AKT, p-AKT, mTOR, and p-mTOR in MNNG/HOS** (F)** and MG63** (G)** cells with HSPD1 overexpression. **(H-I)** Western blot assay revealed the expression of AKT, p-AKT, mTOR, and p-mTOR in HSPD1-silenced MNNG/HOS **(H)** and MG63** (I)** cells with/without AKT activator.** (J-K)** Western blot assay revealed the expression of AKT, p-AKT, mTOR, and p-mTOR in HSPD1-upregulated MNNG/HOS** (J)** and MG63** (K)** cells with/without AKT inhibitor. **(L-M)** Western blot assay revealed the expression of EMT markers in HSPD1-silenced MNNG/HOS** (L)** and MG63** (M)** cells with/without AKT activator.** (N-O)** Western blot assay revealed the expression of EMT markers in HSPD1-upregulated MNNG/HOS** (N)** and MG63** (O)** cells with/without AKT inhibitor.

To elucidate the potential molecular mechanisms by which HSPD1 mediates osteosarcoma progression, a comparison of differentially expressed genes between high and low HSPD1 expression groups was performed, with 109 or 54 genes being up-or down-regulated in the HSPD1-overexpressing cohorts **(Figure S8)**. GO enrichment analysis demonstrated that these genes were significantly associated with ribosome biogenesis, ribonucleoprotein complex biogenesis, chaperone complex, protein folding chaperone, and unfolded protein binding **(Figure 7A)**. GSEA analysis based on HSPD1 transcript expression from all osteosarcoma patients in the training and validation cohorts showed that the positively regulated gene set by mTOR signaling (MTOR_UP.N4.V1_UP) was enriched significantly in HSPD1-overexpressing cohorts **(Figure 7B)**. Inversely, HSPD1 expression was negatively correlated with the AKT_UP.V1_DN gene set **(Figure 7C)**. Following the above evidence, we hypothesized that the AKT/mTOR pathway may be a potential mechanism by which HSPD1 mediates osteosarcoma progression. Prior investigations have indicated that HSPD1 silencing-mediated inactivation of the mTOR pathway led to inhibition of progression in glioblastoma and colorectal cancer (CRC) 57, 58. Mitochondrial unfolded protein response inhibitors targeting HSPD1 induced accumulation of poly-ubiquitinated proteins and metabolic stress, thereby suppressing AKT/mTOR signaling in prostate cancer 59. Moreover, our hypothesis is also supported by the observation that HSPD1 silencing significantly attenuated the phosphorylation levels of AKT and mTOR, without changes in total protein levels **(Figure 7D-E)**. However, Ser473-phosphorylated AKT (p-AKT) and Ser2448-phosphorylated mTOR (p-mTOR) were upregulated by HSPD1 overexpression** (Figure 7F-G)**. Additionally, we treated osteosarcoma cells with the AKT activator SC79 or the inhibitor MK2206, among which shHSPD1#1 was employed for the following experiments, referred to herein as shHSPD1. We observed that phosphorylation levels of AKT and mTOR rose significantly under the AKT activator SC79 treatment, and the reduction of N-cadherin and vimentin resulting from HSPD1 knockdown was partially attenuated in MNNG/HOS and MG63 cells **(Figure 7H-I, L-M)**. MK2206 treatment significantly reduced the levels of p-AKT, p-mTOR, N-cadherin, and vimentin in osteosarcoma cells, while overexpression of HSPD1 partially abrogated these inhibitory effects **(Figure 7J-K, N-O)**. These changes suggest that HSPD1 positively regulates AKT/mTOR signaling, thus mediating EMT of osteosarcoma.

### HSPD1 interacts with ATP5A1 and maintains its stability

**Figure 8:**
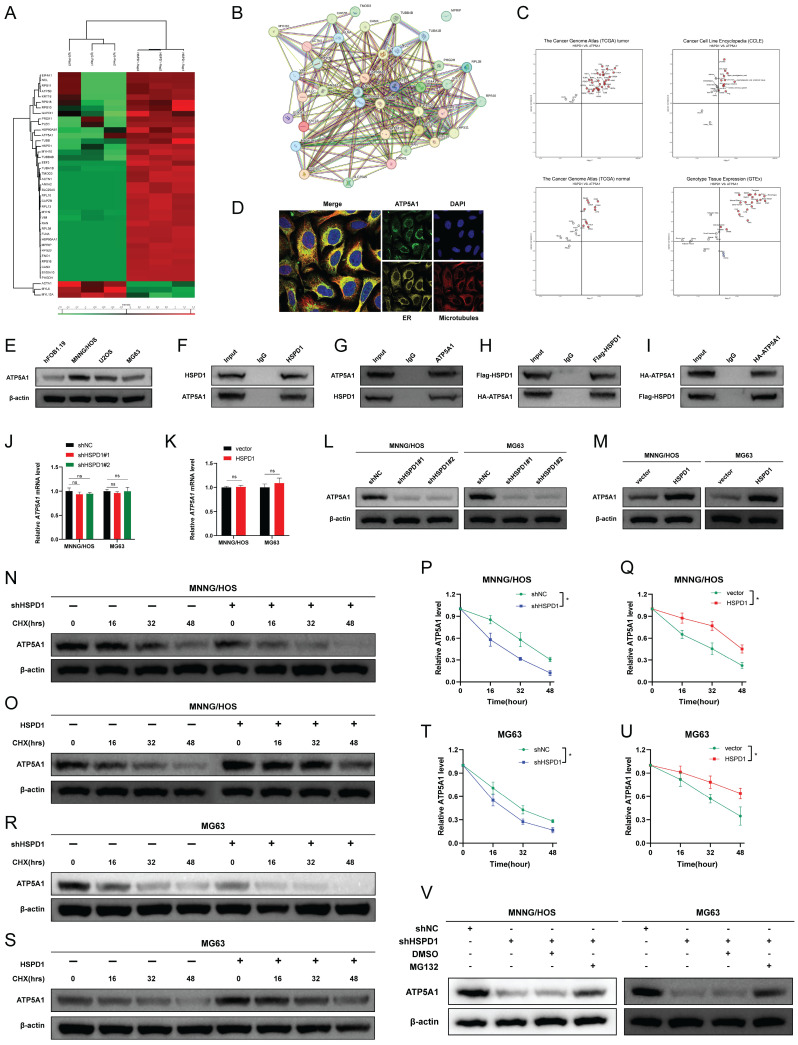
** HSPD1 interacts with ATP5A1 and enhances ATP5A1 protein level. (A)** Identification of HSPD1 partners by co-immunoprecipitation (co-IP) and liquid chromatography-tandem mass spectrometry (LC-MS/MS). MS heatmap showing proteins with a significant change in IP product using antibodies against HSPD1 or IgG (control), with heatmap colors corresponding to log-transformed z-scored iBAQ expression values. **(B)** The protein-protein interactions were generated using the STRING database, based on the thirty-eight HSPD1 hits from the co-IP/MS analysis. **(C)** Co-expression relationship between HSPD1 and ATP5A1 in TCGA pan-cancer datasets, TCGA normal datasets, GTEX datasets, and CCLE datasets. **(D)** Immunofluorescence showed the subcellular location of ATP5A1 in the osteosarcoma cells. **(E)** Expression of ATP5A1 in hFOB1.19 cells and osteosarcoma cell lines detected by Western blot. **(F-G)** Co-IP analysis of the interaction of endogenous HSPD1 and ATP5A1 with anti-HSPD1** (F)** or anti-ATP5A1** (G)** antibodies.** (H-I)** Exogenous protein interactions between HSPD1 and ATP5A1 in HEK 293T cells transfected with Flag-HSPD1** (H)** and HA-ATP5A1 **(I)** plasmid.** (J-K)** qRT-PCR analysis of ATP5A1 after HSPD1 knockdown** (J)** or overexpression** (K)**. **(L-M)** Western blotting analysis of ATP5A1 after HSPD1 knockdown** (L)** or overexpression** (M)**. **(N)** ATP5A1 degradation in HSPD1-silenced MNNG/HOS cells was assessed by cycloheximide (CHX) chase at indicated time points. **(O)** ATP5A1 degradation in HSPD1-upregulated MNNG/HOS cells was assessed by CHX chase at indicated time points. **(P-Q)** Quantification of protein degradation kinetics of ATP5A1 in HSPD1-silenced **(P)** or upregulated **(Q)** MNNG/HOS cells. **(R)** ATP5A1 degradation in HSPD1-silenced MG63 cells was assessed by CHX chase at indicated time points. **(S)** ATP5A1 degradation in HSPD1-upregulated MG63 cells was assessed by CHX chase at indicated time points. **(T-U)** Quantification of protein degradation kinetics of ATP5A1 in HSPD1-silenced **(T)** or upregulated **(U)** MG63 cells. **(V)** Western blotting showed that MG132 reversed HSPD1 knockdown-induced ATP5A1 degradation in MNNG/HOS and MG63 cells. *P < 0.05; **P < 0.01; ***P < 0.001.

To further investigate the underlying mechanism of HSPD1 in osteosarcoma, HSPD1-interacting proteins were characterized by co-immunoprecipitation/mass spectrometry (coIP/MS) analysis. A total of 38 potential targets were identified and further imported into the STRING database **(Figure 8A)**. ATP5A1, also named as ATP5F1A, was predicted to be one of the major partners of HSPD1 in the STRING-identified protein-protein interaction (PPI) network **(Figure 8B)**. Furthermore, Pearson's correlation analysis revealed that HSPD1 was positively associated with ATP5A1 in most normal and cancer tissues or cell lines based on the data from GTEx, TCGA, and CCLE databases **(Figure 8C)**. Additionally, immunofluorescence results demonstrated that the majority of ATP5A1 was localized in the mitochondria, with barely any signal observed in the nucleus **(Figure 8D)**. Comparing ATP5A1 expression in normal osteoblasts (hFOB1.19) against each of the three different osteosarcoma cells (U2OS, MG63, and MNNG/HOS) revealed upregulation of the gene in tumor **(Figure 8E)**.

We hypothesized that HSPD1 might bind to ATP5A1 in osteosarcoma cells, which was further confirmed by coIP protein blotting. Endogenous HSPD1 coprecipitated with ATP5A1, and ATP5A1 was also efficiently coimmunoprecipitated with HSPD1 in MNNG/HOS cells **(Figure 8F-G)**. Furthermore, as shown in **Figure 8H-I**, exogenous HSPD1 and ATP5A1 could also be coimmunoprecipitated in HEK-293T cells. In conclusion, the results we have presented so far indicate that HSPD1 could interact with ATP5A1. In addition, HSPD1 knockdown or overexpression had no effect on ATP5A1 mRNA expression levels **(Figure 8J-K)**. However, HSPD1 silencing significantly reduced ATP5A1 protein levels, with the reverse result for HSPD1 up-regulation **(Figure 8L-M)**. Ubiquitination is a crucial process for maintaining mitochondrial homeostasis and mitochondrial quality control. Previous studies have demonstrated that HSPD1 silencing could facilitate proteasome-mediated degradation of mitochondrial 3-oxoacyl-ACP synthase 46. Based on these findings, we postulated that HSPD1 downregulation may play a role in mediating the loss of ATP5A1 protein stability and subsequent degradation. CHX chase experiments were performed to evaluate whether HSPD1 alters endogenous ATP5A1 expression by regulating protein degradation. The half-life of ATP5A1 protein was shorter in the HSPD1 knockdown group, whereas the degradation rate was notably slowed after HSPD1 overexpression **(Figure 8N-U)**. This degradation was blocked by the proteasome inhibitor MG132, suggesting that the ubiquitin protease system was involved **(Figure 8V)**.

### HSPD1 silencing induces the ATP5A1 K48-linked ubiquitination and proteasomal degradation in osteosarcoma

**Figure 9:**
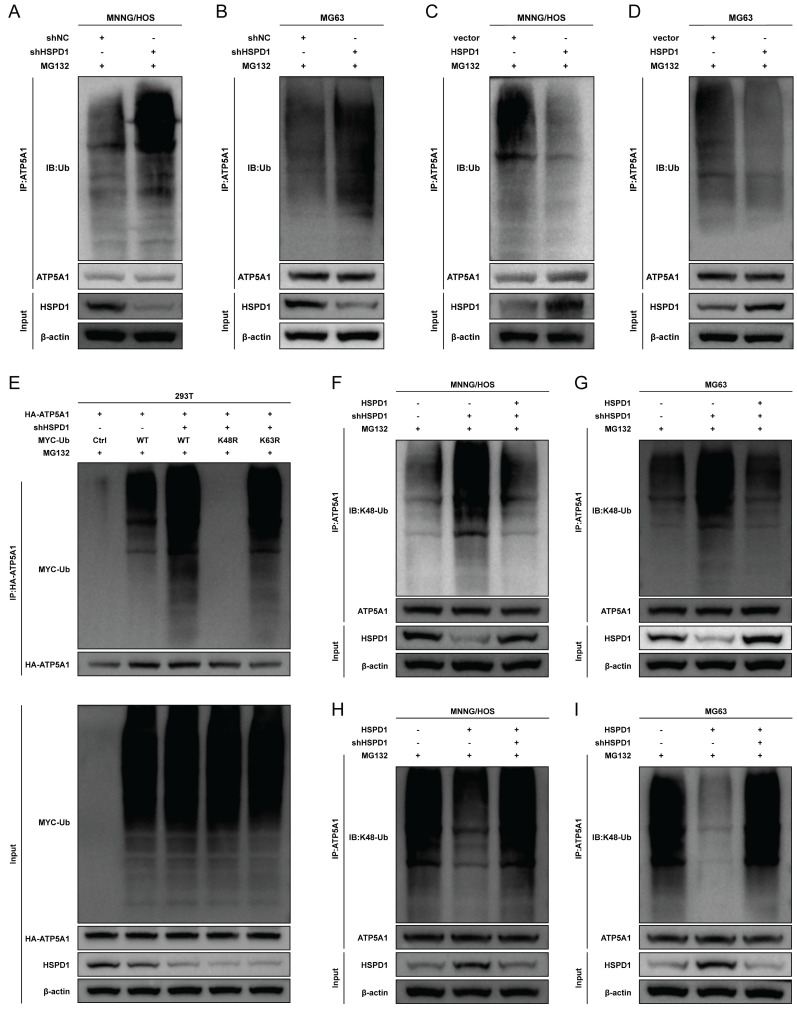
** HSPD1 silencing promotes the K48-linked ATP5A1 ubiquitination and degradation. (A-B)** In the presence of MG132, the ubiquitination level of ATP5A1 in HSPD1-silenced osteosarcoma cells was detected by co-IP.** (C-D)** In the presence of MG132, the ubiquitination level of ATP5A1 in HSPD1-upregulated osteosarcoma cells was detected by co-IP. **(E)** In the presence of MG132, the shHSPD1, HA-ATP5A1, MYC-Ub, and ubiquitin mutant plasmids (only one lysine residue was mutated to an arginine residue) were co-transfected into 293T cells for ubiquitination assays. **(F-G)** Enforced HSPD1 expression reduced the K48-linked ubiquitination of ATP5A1 in HSPD1-silencing osteosarcoma cells. **(H-I)** HSPD1 knockdown enhanced the K48-linked ubiquitination of ATP5A1 in HSPD1-upregulated osteosarcoma cells.

Next, we tested the effect of HSPD1 on ATP5A1 ubiquitination and observed that HSPD1 overexpression inhibited ATP5A1 polyubiquitination, whereas HSPD1 depletion increased ATP5A1 ubiquitination levels in osteosarcoma cells **(Figure 9A-D)**. These results suggest that HSPD1 affects ATP5A1 ubiquitination and stabilizes ATP5A1. However, the type of ubiquitin chain produced on ATP5A1 is still unknown.

Polyubiquitin linkages via lysine 48 (K48) or lysine 63 (K63) could differentially address proteins for proteasomal degradation or endosome trafficking to the lysosome 60. To explore the type of ubiquitin chain generated on ATP5A1, we mutated the lysine residues on ubiquitin and evaluated their effect on ATP5A1 ubiquitination. It was found that the K48R (Lys48 mutated to Arg) mutation in ubiquitin completely prevented ATP5A1 polyubiquitination in 293T cells** (Figure 9E)**. Furthermore, while HSPD1 silencing enhanced ATP5A1 K48-linked ubiquitination to reduce the levels of ATP5A1 protein, HSPD1 over-expression had the opposite effect in osteosarcoma cells **(Figure 9F-I)**. More importantly, the re-introduction of HSPD1 expression significantly attenuated the ATP5A1 K48-linked ubiquitination in the HSPD1-silenced osteosarcoma cells, while HSPD1 silencing obviously rescued the ATP5A1 K48-linked ubiquitination in the HSPD1-overexpressing osteosarcoma cells **(Figure 9F-I)**. Taken together, we confirmed that the knockdown of HSPD1-mediated ATP5A1 ubiquitination is K48-linked.

### ATP5A1 promotes the phosphorylation of mTOR via upregulation of ATP generated from OXPHOS

**Figure 10:**
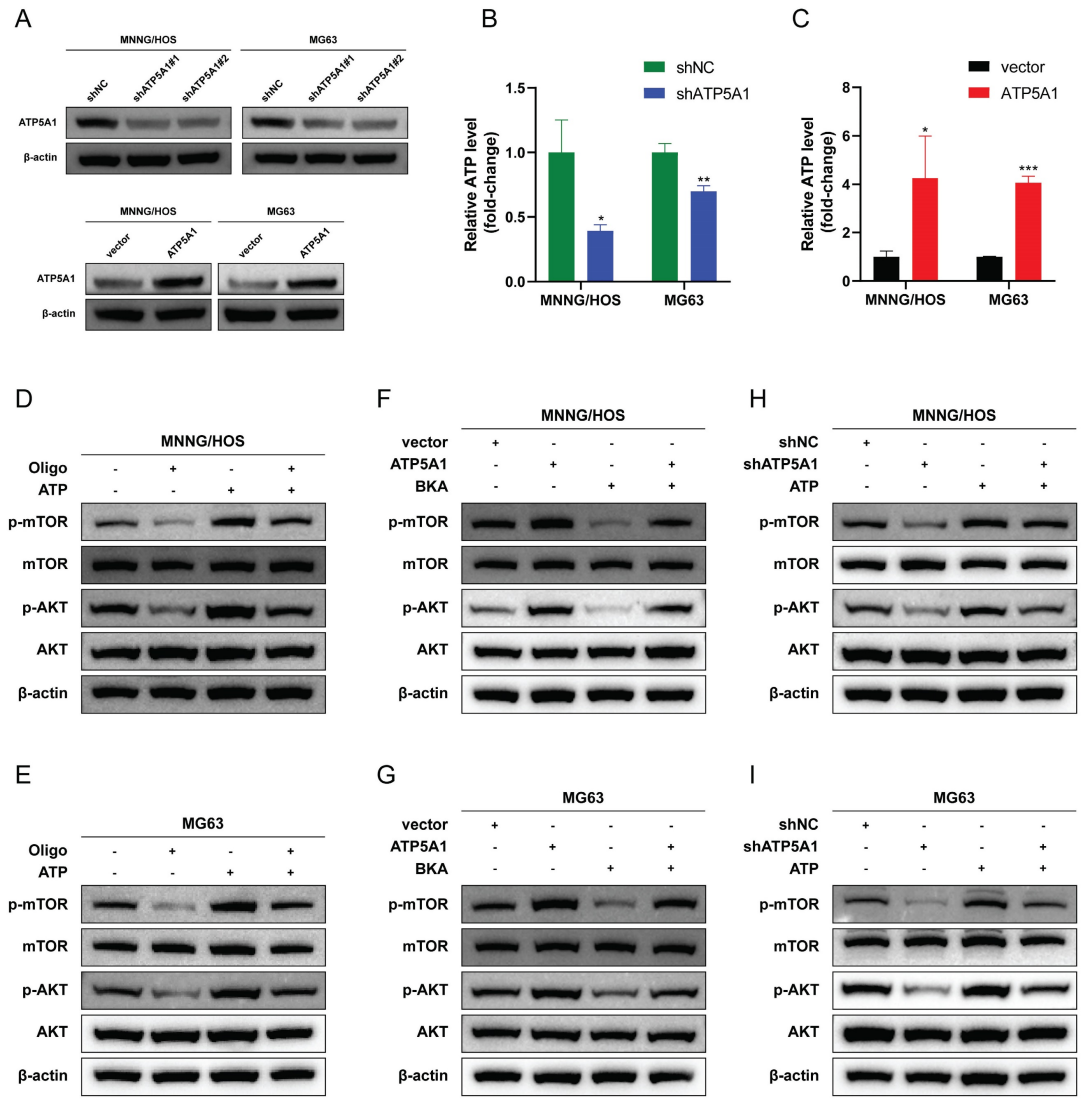
** Inhibition of ATP5A1 attenuates AKT/mTOR signaling via decreased mitochondrial ATP generation. (A)** The efficiency of ATP5A1 overexpression and knockdown were verified through western blot.** (B-C)** ATP production was assessed by ATP assay kit (Beyotime) in MNNG/HOS and MG63 cells transfected with ATP5A1-specific shRNA or ATP5A1 overexpression vector. The ATP level was normalized to the protein concentration. **(D-E)** Treatment with mitochondrial OXPHOS complex inhibitors oligomycin (Oligo, 2 μM) for 48 hours downregulated p-AKT and p-mTOR expression in osteosarcoma cells. Pretreatment of cells with 2 mM ATP rescued the expression of p-AKT and p-mTOR.** (F-G)** Treatment with 10 μM adenine nucleotide translocator (ANT) inhibitor bongkrekic acid (BKA) for 48 hours inhibited the expression of p-AKT and p-mTOR in ATP5A1-upregulated osteosarcoma cells. **(H-I)** ATP enhanced the expression of p-AKT and p-mTOR in ATP5A1-silencing osteosarcoma cells. The data are presented as mean ± SD. *P < 0.05; **P < 0.01; ***P < 0.001.

We next sought to explore the significance of ATP5A1 protein homeostasis in osteosarcoma cells. The production of ATP in mitochondria is dependent on the electron transport chain and OXPHOS 61. ATP5A1 is a subunit of mitochondrial respiratory chain complex V, which controls ATP production from ADP in the presence of a proton gradient across the inner membrane 62, 63. We used two shRNAs targeting different domains of the ATP5A1 mRNA sequence to transfect osteosarcoma cells to avoid off-target effects. ATP5A1 protein levels were all drastically reduced **(Figure 10A)**, so the shRNA pool (shATP5A1) was used for subsequent experiments. As shown in **Figure 10B-C**, ATP5A1 silencing resulted in reduced ATP production, indicating that OXPHOS was strongly inhibited, whereas ATP5A1 overexpression promoted mitochondrial ATP production in osteosarcoma cells. These results suggest that ATP5A1 affects the homeostatic level of ATP in osteosarcoma.

Reduced ATP generation could regulate mTOR at multiple levels and inhibit mTOR signaling 64. Therefore, we sought to investigate whether ATP5A1 supports mTOR signaling activation by regulating the homeostatic level of ATP. The classical ATP synthase inhibitor oligomycin significantly inhibited mTOR signaling, suggesting that ATP produced by OXPHOS is essential for mTOR activation **(Figure 10D-E)**. As shown in **Figure 10F-G**, ATP5A1 overexpression resulted in increased levels of p-AKT and p-mTOR in osteosarcoma cells. Bongkrekic acid (BKA), an inhibitor of the adenine nucleotide translocator (ANT), antagonizes ATP translocation from mitochondria into the cytosol to reduce cytoplasmic ATP/ADP ratios 65, 66. Notably, BKA blocked the effect of ATP5A1 overexpression on mTOR signaling in osteosarcoma cells, a finding that indicates the ATP generated by ATP5A1 overexpression is indispensable for mTOR activation **(Figure 10F-G)**. Similarly, ATP5A1 knockdown impaired mTOR phosphorylation in osteosarcoma cells, whereas ATP supplementation partially restored mTOR signaling **(Figure 10H-I)**. These results suggest that ATP5A1 maintains OXPHOS function and promotes mitochondrial ATP production, which in turn promotes mTOR activation in osteosarcoma cells.

### ATP5A1 mediates the oncogenic properties of HSPD1 in osteosarcoma

**Figure 11:**
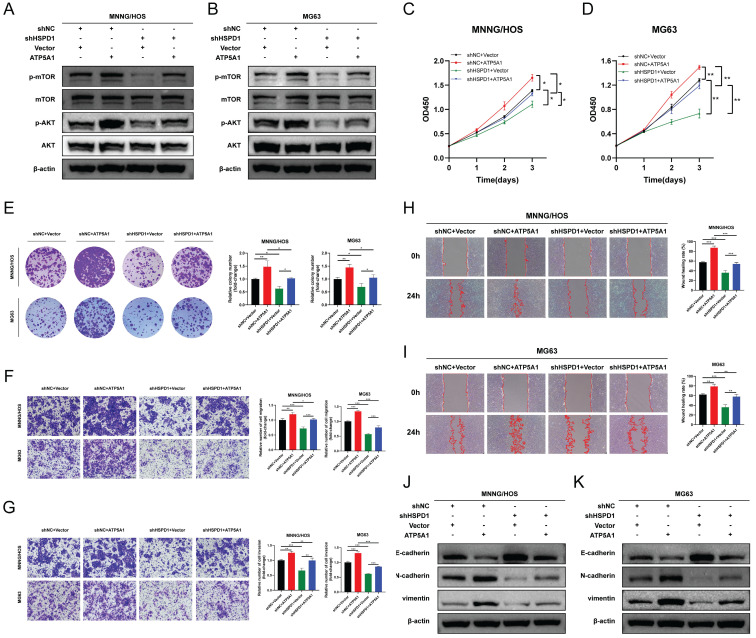
** HSPD1 regulates osteosarcoma progression in an ATP5A1-dependent manner. (A-B)** Western blot assay revealed the expression of AKT, p-AKT, mTOR, and p-mTOR in MNNG/HOS** (A)** and MG63** (B)** cells transfected with HSPD1 specific shRNA and/or ATP5A1 overexpression vector. **(C-D)** Cell proliferation was assessed by CCK-8 assay in MNNG/HOS **(C)** and MG63** (D)** cells transfected with HSPD1 specific shRNA and/or ATP5A1 overexpression vector (shNC+Vector, shNC+ATP5A1, shHSPD1+Vector, shHSPD1+ATP5A1). **(E)** The clonogenic potential was assessed by colony formation assay in MNNG/HOS and MG63 cells transfected with HSPD1-specific shRNA and/or ATP5A1 overexpression vector. **(F)** Cell migration was assessed by transwell assay in MNNG/HOS and MG63 cells transfected with HSPD1-specific shRNA and/or ATP5A1 overexpression vector.** (G)** Cell invasion was assessed by transwell assay in MNNG/HOS and MG63 cells transfected with HSPD1-specific shRNA and/or ATP5A1 overexpression vector.** (H-I)** Cell migration was assessed by wound healing assay in MNNG/HOS** (H)** and MG63 **(I)** cells transfected with HSPD1 specific shRNA and/or ATP5A1 overexpression vector.** (J-K)** Western blot assay revealed the expression of EMT markers in MNNG/HOS** (J)** and MG63 **(K)** cells transfected with HSPD1 specific shRNA and/or ATP5A1 overexpression vector. The data are presented as mean ± SD. *P < 0.05; **P < 0.01; ***P < 0.001.

We then asked whether ATP5A1 was involved in HSPD1-induced overactivation of the AKT/mTOR axis and the corresponding phenotypic changes in osteosarcoma. The effect of ATP5A1 on the activation of mTOR signaling by HSPD1 was examined. Expression levels of p-AKT and p-mTOR were increased upon ATP5A1 upregulation, and importantly, the inhibition of phosphorylation levels of biomarkers within the mTOR pathway in HSPD1 knockdown osteosarcoma cells was partially reversed by exogenous expression of ATP5A1** (Figure 11A-B)**. We further investigated the involvement of ATP5A1 in the mechanism of HSPD1-promoted osteosarcoma progression using several *in vitro* functional rescue assays. Cell proliferation assays showed that ATP5A1 overexpression promoted cell viability and colony-forming ability of MNNG/HOS and MG cells and partially reversed HSPD1 knockdown-mediated inhibition of cell proliferation **(Figure 11C-E)**.

A similar trend was observed in the transwell and scratch wound healing assays **(Figure 11F-I)**. The above results showed that the HSPD1 silencing-induced reduction of osteosarcoma malignant behaviors such as proliferation and invasion was partially counteracted by ATP5A1 overexpression. At the same time, ATP5A1 overexpression prevented HSPD1 downregulation-mediated EMT inhibition **(Figure 9J-K)**. These results reveal the essential function of ATP5A1 for HSPD1 promoting the AKT/mTOR pathway activation in osteosarcoma.

### HSPD1 depletion impairs tumorigenesis of osteosarcoma *in vivo*

**Figure 12:**
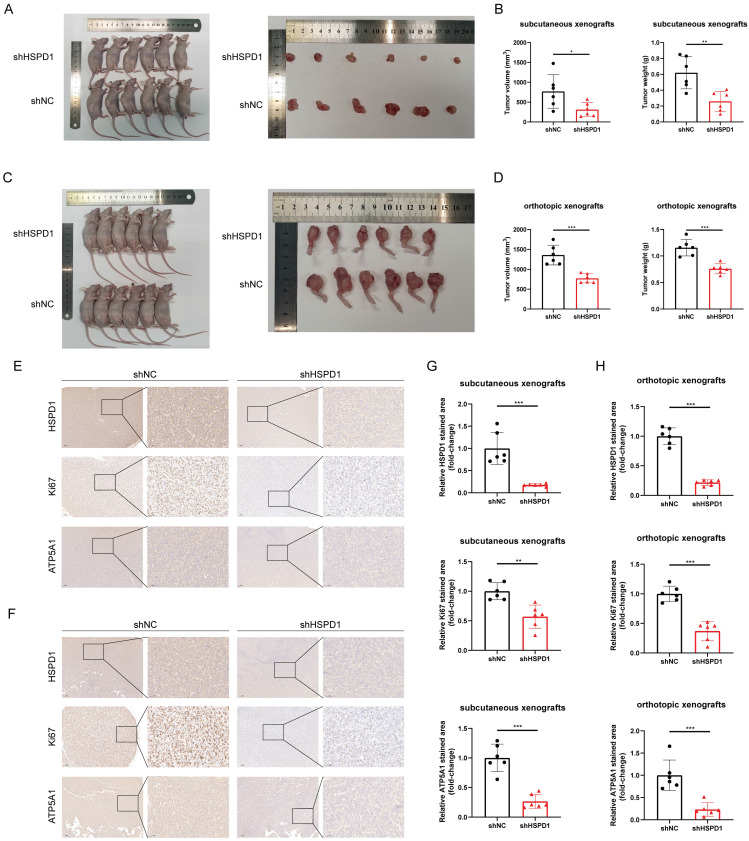
** HSPD1 depletion impairs tumorigenesis of osteosarcoma *in vivo*. (A)** Macrograph of xenograft tumors after subcutaneous injection of MNNG/HOS cells with or without HSPD1 knockdown.** (B)** The volume and weight of subcutaneous xenograft tumors after knockdown of HSPD1.** (C)** Macrograph of xenograft tumors after orthotopic injection of MNNG/HOS cells with or without HSPD1 knockdown.** (D)** The volume and weight of orthotopic xenograft tumors after knockdown of HSPD1.** (E)** Immunohistochemical (IHC) analysis of HSPD1, Ki67, and ATP5A1 in subcutaneous xenograft tumors derived from the shNC and shHSPD1 groups. Montage scale bar, 100μm; magnified-view scale bar, 50μm.** (F)** IHC analysis of HSPD1, Ki67, and ATP5A1 in orthotopic xenograft tumors derived from the shNC and shHSPD1 groups. Montage scale bar, 100μm; magnified-view scale bar, 50μm.** (G)** Semiquantitative analysis of IHC staining for HSPD1, Ki67, and ATP5A1 in subcutaneous xenograft models of osteosarcoma.** (H)** Semiquantitative analysis of IHC staining for HSPD1, Ki67, and ATP5A1 in orthotopic xenograft models of osteosarcoma. The data are presented as mean ± SD. *P < 0.05; **P < 0.01; ***P < 0.001.

To assess the impact of HSPD1 on osteosarcoma growth *in vivo*, MNNG/HOS cells with suppressed HSPD1 were created and injected into Balb/c nude mice to establish subcutaneous and orthotopic osteosarcoma models with six mice in each group. As shown in **Figure 12A-D, and Figure S9A**, tumors derived from shHSPD1 exhibited significant volume and weight inhibition in both subcutaneous and orthotopic xenograft models compared to shNC-derived control xenografts. IHC analysis revealed a notable decrease in the percentage of Ki-67-positive cancer cells in the shHSPD1 group when compared to the control group **(Figure 12E-H)**. Analysis of ATP5A1 and EMT-related markers in xenografts revealed that reducing HSPD1 led to lower levels of ATP5A1, vimentin, and N-cadherin, while increasing E-cadherin expression **(Figure 12E-H, Figure S9B-E)**. These findings support that HSPD1 knockdown suppresses the advancement of osteosarcoma *in vivo*.

## Discussion

HSPs, which are expressed during stressful conditions or carcinogenesis, are highly conserved, widely distributed, and abundant 67. They exert diversified biological roles, such as overseeing and aiding in protein folding, preventing protein clumping, aiding in the restoration of denatured proteins, assisting in the movement of proteins across membranes, and contributing to protein breakdown processes 68. During tumorigenesis, HSPs have been central targets in regulating aspects of tumor cell response and malignant phenotypes, with genomes across cancers containing augmented HSPs 50, 69. Certain crucial HSPs also intricately control the equilibrium of defensive and harmful immune reactions in the TME 70. As one of the most lethal tumors in children and adolescents, osteosarcoma is characterized by high aggressiveness and mortality 71. Extensive research currently delves into the potential role of individual HSPs such as HSPB1, HSPBP1, and HSP90AA1 in influencing the survival and advancement of osteosarcoma, yet the prognostic value and molecular mechanisms of other HSP family members in osteosarcoma remain to be elucidated 72-77. Although more data are needed to clarify the impact of HSPD1 expression on osteosarcoma progression and the immune microenvironment, our study describes for the first time that HSPD1 inhibits K48 ubiquitination degradation of ATP5A1, which facilitates mitochondrial ATP production and activation of AKT/mTOR signaling.

Initially, we conducted a comprehensive analysis of the impact of HSP family members on prognostic and TME cell infiltration characteristics in osteosarcoma using multi-omics data. An unsupervised hierarchical clustering analysis was implemented on the patient population, based on twenty-four prognostic HSPs derived from univariate Cox regression analysis. We revealed two distinct HSP modification patterns and noted contrasting biological functions between them. However, due to the genomic heterogeneity and complexity in individual osteosarcoma patients, an HSP-derived signature was constructed in addition to the above population-based cohort study. HSP-derived signature provides an effective classification of osteosarcoma patients into low and high-risk groups and adds prognostic value to traditional clinicopathologic risk factors by nomogram. ROC curves assessed the predictive precision of HSPscore for 1-, 3-, and 5-year overall survival in osteosarcoma, demonstrating excellent specificity and sensitivity. High hazard ratios indicated that HSPscore served as an independent unfavorable prognostic factor, based on both univariate and multivariate Cox regression analysis. In this analysis, we emphasized the variations in tumor-immune microenvironment (TIME) among the high and low HSPscore categories. The increased presence of CD8+ T cells, DCs, macrophages, mast cells, neutrophils, NK cells, pDCs, T helper cells, Tfh cells, Th2 cells, TILs, and Tregs were detected in the low HSPscore group, indicating a better prognosis for patients. The immunoinflammatory phenotype, called hot tumors, is manifested by a large infiltration of immune cells in the TME 78-80. Cold tumors with low immunogenicity exhibit immunosuppressive mechanisms that resist immunotherapy 81. In line with the definition provided, the low HSPscore group displayed immune activation and high levels of immune cell infiltration, indicating an immunoinflammatory phenotype; on the other hand, the high HSPscore group showed immune suppression, indicating an immune-desert phenotype. It is therefore not surprising that the low HSPscore group showed a significant survival advantage in the survival analysis. Previous reports have indicated that NK cells and CD8+ T cells play a significant role in the antitumor response, with their quantity and activity level being key factors in determining the effectiveness against tumors 81-83. With continued exposure to antigens and activation by inflammatory substances, CD8+ T cells progressively diminish in their ability to proliferate and release effector cytokines 84. Exhausted CD8+ T cells exhibit a unique transcriptome compared to memory and effector CD8+ T cells, which is a significant barrier to the success of cancer immunotherapy 85. There may be a positive correlation between the presence of tumor-infiltrating NK cells and prolonged patient survival 86. During the initial phases of the immune response against tumors, NK cells are first attracted to the tumor microenvironment, where they are subsequently stimulated by various ligand-receptor interactions to collaboratively impede tumor progression along with T cells 87. Current preclinical and clinical development strategies include the use of adoptive transfer therapies, recruitment of NK cells to the TME, direct stimulation, and blockade of inhibitory receptors to enhance the NK cell-mediated killing of cancer cells 88, 89. Similar to NK cells, tumor-associated neutrophils impair cancer progression through direct cytotoxic effects or activation of innate or adaptive immunity 90. Several other cell types, such as DCs, TILs, and Tregs also gained considerable attention for their complex roles in tumorigenesis 91. Notably, we observed a notable reduction in the abundance of CAFs in the high HSPscore group (patients with poorer prognosis) using the EPIC and MCPCounter algorithms. There is mounting evidence that CAFs possess antitumor immune functions and that CAF depletion also has a detrimental impact on outcomes in preclinical models 92. In conclusion, it could be postulated that the favorable prognosis observed in the low HSPscore group may be attributed to its immune activation; whereas the high HSPscore suggests that the infiltration levels of various key antitumor immune cells are inhibited, leading to weakened antitumor immune response within the TME.

ICIs (immune checkpoint inhibitors) have been employed as a primary therapeutic approach for a range of malignant neoplasms, working by blocking specific inhibitory receptors on immune cells and improving their ability to detect and fight against cancerous cells 93. ICIs, particularly targeted modulation of the PD-1/PD-L1 and CTLA-4 inhibitory pathways, have become a primary area of study in addressing immunosuppression caused by osteosarcoma 94, 95. Previous studies have demonstrated a positive correlation between ICG expression levels and immune cell infiltration in tumors 96. Additionally, higher expression of activated ICGs has been associated with a more favorable response of patients to immune checkpoint blockade (ICB) immunotherapy 97. A potential mode of resistance to ICIs involves the aberrant HLA antigen presentation pathway, which determines the specificity of CD8+ T cells and is critical for their activation and proliferation 98. This study demonstrated that individuals in the low HSPscore group displayed considerably elevated checkpoint and HLA expression levels compared to the high HSPscore group, indicating a potential responsiveness to immunotherapy with ICIs. Subsequent analyses further validated the detailed relationship between HSPscore and clinicopathologic features, including survival and metastatic status. Furthermore, we confirmed the predictive value of HSPscore for drug sensitivity, which could provide new insights into drug screening for osteosarcoma. Collectively, our prognostic model may prove beneficial for patient counseling and the implementation of more personalized management strategies for osteosarcoma patients, thereby offering innovative insights into the prediction of TME landscape features and ICI treatment responses.

Among the four pivotal HSPs, Kaplan-Meier curves revealed that osteosarcoma patients with high expression levels of DNAJC5B and DNAJC17 exhibited longer OS, though the differences were not statistically significant. Conversely, individuals with elevated levels of HSPD1 and DNAJC1 experienced inferior outcomes. DNAJC1, DNAJC5B, and DNAJC17 are members of the DNAJ (HSP40) chaperone family, which cooperates with HSP70 molecular chaperones to regulate the proper folding of other proteins 99-101. The differential expression of DNAJ proteins in human tissues suggests a potential role for DNAJ isoforms in the development and spread of cancer by serving as co-chaperones for various oncogenes or tumor suppressors 100. DNAJC1 was upregulated in most malignant tumors (including HCC) and may be involved in cancer initiation and development 102. The oncogenic properties of DNAJC1 in HCC cells may be attributed to its regulation of the p53 and EMT pathways, leading to increased proliferation, migration, invasion, and suppression of apoptosis 102. DNAJC1 has been thoroughly examined in relation to stress and immune reactions, specifically in its function of controlling the helper T cell phenotype in thyroid tissue 103. DNAJC5B has been identified as an intracellular factor that inhibits hepatitis C virus replication and may serve as a prognostic biomarker for esophageal squamous cell carcinoma 104, 105. DNAJC17 is found in nuclear speckles and interacts with components of the splicing machinery, leading to increased splicing efficiency in HeLa cells 101. Our discovery aligns with prior research conducted by Chen *et al.*, indicating that elevated levels of DNAJC17 are linked to a positive prognosis for individuals with osteosarcoma 106.

Notably, HSPD1 is upregulated in osteosarcoma tissues and cell lines and may serve as an independent prognostic marker. Accordingly, the present study is dedicated to elucidating the specific function and mechanism of HSPD1 in osteosarcoma. Recent studies have demonstrated that HSPD1 plays a pivotal role in the protein quality control system 107. Normal cells activate mitochondrial unfolded protein response to maintain mitochondrial protein homeostasis, leading to cellular homeostasis and health 108, 109. Cancer cells, on the other hand, hijack this unique pathway to promote their long-term survival, leading to cancer progression and metastasis 59, 110. Our findings indicate that the overexpression of HSPD1 may facilitate osteosarcoma progression by promoting cell proliferation, colony formation, and metastasis while disrupting apoptosis. Conversely, silencing of HSPD1 leads to the opposite effect. These results suggest that the oncogenic role of HSPD1 in osteosarcoma may be analogous to the results of previous studies on oral squamous cell carcinoma, gastric cancer, CRC, pancreatic cancer, and liver hepatocellular carcinoma 41, 111-114. EMT is a critical process in tumor cell invasion and migration during cancer development 115. Regulation of the EMT process involves an intricate system of signaling pathways and transcription factors, which are characterized by the upregulation of Vimentin and N-cadherin 116, 117. In this study, Western blot confirmed that knockdown of HSPD1 in osteosarcoma cells triggered a decrease in Vimentin and N-cadherin and an upregulation of E-cadherin. These results indicate that HSPD1 silencing may inhibit EMT, thereby suppressing osteosarcoma cell metastasis and invasion. Concurrently, the absence of HSPD1 demonstrated a robust capacity to diminish tumorigenesis of osteosarcoma cells *in vivo*. Collectively, the *in vivo* and *ex vivo* outcomes indicated that HSPD1 facilitated osteosarcoma cell proliferation.

It has been documented that HSPD1 influences a number of cancer-related signaling pathways, including the NF-κB, MAPK, ERK, and AKT/mTOR pathways 41, 59, 118, 119. However, the precise mechanism by which HSPD1 regulates the progression of osteosarcoma remains unclear. GSEA analysis based on HSPD1 transcript expression in osteosarcoma cohorts revealed that the gene set positively regulated by mTOR signaling (MTOR_UP.N4.V1_UP) was significantly enriched in the HSPD1-overexpressing cohorts, indicating that mTOR signaling is activated. The PI3K/AKT/mTOR is one of the numerous signaling cascades that govern multiple cellular and molecular processes fundamental to tumor initiation, invasion, and metastasis 120. Its constituent genes have been extensively studied and found to be commonly activated in human cancers. As an important cell cycle mediator, AKT is often highly activated by phosphorylation at Thr308 and Ser473, promoting cancer cell proliferation and migration and resisting apoptosis 121-123. Previous studies have also demonstrated a strong link between HSPD1 and the AKT/mTOR pathway, e.g., HSPD1 silencing-mediated inactivation of the mTOR pathway inhibited the progression of glioblastoma and CRC 57, 58. Inhibitors of the mitochondrial unfolded protein response targeting HSPD1 induced accumulation and metabolic stress of polyubiquitinated proteins, thereby inhibiting AKT/mTOR signaling in prostate cancer 59. Thus, we hypothesized that the AKT/mTOR pathway could serve as a potential mechanism through which HSPD1 facilitates the advancement of osteosarcoma. The hypothesis is supported by the observation that HSPD1 overexpression enhanced mTOR phosphorylation and activated the mTOR signaling pathway. However, HSPD1 silencing partially abolished this effect, that is, reversion of function occurred, consistent with evidence that HSPD1 is an upstream regulator of the AKT/mTOR pathway. As important downstream targets of the AKT/mTOR pathway, the effects of increased phosphorylation of AKT and mTOR on the proliferation, migration, and apoptosis in various malignancies are well known 120, 124. Furthermore, we demonstrated the effects of HSPD1 on key downstream metastasis-related effectors of the AKT signaling pathway, including Vimentin, N-cadherin, and E-cadherin. These results suggest that the oncogenic function of HSPD1 in osteosarcoma proliferation and EMT depends, to some extent, on the stimulation of the AKT/mTOR pathway.

At the mechanistic level, our findings indicate that HSPD1 may interact with ATP5A1 and increase ATP5A1 protein levels, thereby activating the AKT/mTOR pathway to mediate osteosarcoma progression. ATP synthase (complex V) is the crucial final step of OXPHOS. Dysregulation of mitochondrial ATP synthase has been widely reported to be associated with tumorigenicity, tumor metastasis, metabolism, Ca2+ homeostasis, endoplasmic reticulum stress, and chemoresistance 125. ATP5A1, a subunit of mitochondrial ATP synthase, is used as an indicator for mitochondrial complex V 126. Increased expression of ATP5A1 enhances ATP synthase function and reduces mitochondrial superoxide generation and oxidative stress 127. Co-expression analysis revealed that ATP5A1 was overexpressed in the oxidative phosphorylation pathway and was associated with the progression of clear cell renal cell carcinoma 128. The study reported that targeting ATP5A1 was effective in disrupting tumor cells and microvascular proliferation in glioblastoma 129. Conversely, the absence of ATP5A1 has been observed to enhance proliferation, which is indicative of poor progression and an advanced tumor stage in colon adenocarcinoma 130. Despite these findings, the specific details of ATP5A1 degradation remain poorly understood. The ubiquitin-proteasome system is a crucial pathway for selective protein degradation in eukaryotes, with a wide range of physiological functions, including apoptosis, antigen presentation, and intracellular signaling 131. In this study, we demonstrated that HSPD1 hinders the degradation of the mitochondrial protein ATP5A1 through interaction and inhibition of ubiquitination, as evidenced by LC-MS/MS and co-IP experiments. You *et al.* demonstrated that GADD45A affects the ubiquitination degradation of ATP5A1 in adipocytes, which describes another regulatory pathway of ATP5A1 post-translational modification 63. Similarly, previous studies have confirmed that Poly(GR) increases ATP5A1 ubiquitination and degradation, which is a major driver of disease initiation in C9ORF72-related amyotrophic lateral sclerosis and frontotemporal dementia 62. However, the type of ubiquitin chain produced on ATP5A1 is still unknown. These findings indicate that ATP5A1 regulation may be intricately linked to the proteasome pathway. Polyubiquitin linkages via K48 or K63 could differentially address proteins for proteasomal degradation or endosome trafficking to the lysosome 60. We confirmed that the knockdown of HSPD1-mediated ATP5A1 ubiquitination is K48-linked but not K63-linked. Furthermore, we conducted a review of the crosstalk between ATP5A1 and mTOR in cancer. Wang *et al.* investigated that the MTA1-ATP5A1 axis mediates the mTOR pathway by increasing OXPHOS and ATP production, and suppressing ATP5A1 enhances the sensitivity of metastatic colon cancer to the mTOR inhibitor in an MTA1-dependent manner 132. This study proposes that ATP5A1 promotes AKT/mTOR signaling by maintaining ATP generation. Metabolic reprogramming is a hallmark of cancer 133. Functional OXPHOS is essential for supporting cancer cell growth through selective activation of cancer-related mitochondrial retrograde signaling 59, 134. ATP is an important signaling molecule, and sustained OXPHOS function and subsequent ATP generation are critical in tumor progression 64, 135. An elevated ATP/ADP ratio regulates mTOR at multiple levels and promotes mTOR signaling 64, 136, 137. Conversely, disruption of mitochondrial OXPHOS inhibits mTOR kinase activity 138. We demonstrated that ATP5A1 knockdown resulted in reduced ATP production and impaired mTOR phosphorylation in osteosarcoma cells, whereas ATP supplementation partially restored mTOR signaling. Moreover, BKA blocked the effect of ATP5A1 overexpression on mTOR signaling in osteosarcoma cells. Further, *in vitro* functional rescue experiments and WB assays have shown that HSPD1 promotes the proliferation and invasion of osteosarcoma cells through ATP5A1-mediated activation of mTOR signaling. Overall, our results suggest that the knockdown of HSPD1 accelerates K48-linked polyubiquitination degradation of ATP5A1, leading to reduced OXPHOS-generated ATP, which ultimately affects the activation of the downstream mTOR signaling pathway.

Despite our findings on the oncogenic properties of HSPD1 in osteosarcoma, there is still much to be explored in the future. Firstly, although we proposed an HSP-associated signature based on HSPD1, DNAJC1, DNAJC5B, and DNAJC17 and systematically evaluated its value in characterizing the immune landscape of osteosarcoma, further *in vitro* and *ex vivo* experiments are required to validate the role of these genes in regulating the TIME. Secondly, given the dearth of immunotherapy data in the existing retrospective osteosarcoma cohort, further collection of information on osteosarcoma patients treated with ICB, including age, gender, genetic polymorphisms, tumor antigenicity, MHC antigen expression, the function of interferon signaling pathways, and oncogenic signaling pathways, may prove beneficial in exploring the potential value of the HSPscore in predicting the sensitivity to ICB treatment. This is the direction of our future research.

## Conclusions

In conclusion, our analysis of multi-omics data from osteosarcoma patients identified two subtypes based on HSPs that exhibited significant heterogeneity in terms of functional pathways. The HSP-derived scoring system furnishes valuable insights into the tumor-infiltrating immune cell characteristics and clinical perspectives of individual osteosarcoma patients, thereby guiding the development of more effective immunotherapeutic strategies. HSPD1 is aberrantly upregulated in osteosarcoma and associated with shorter OS times. Furthermore, our study provides both *ex vivo* and *in vivo* evidence of the oncogenic properties of HSPD1 in osteosarcoma. Mechanistically, HSPD1 has been demonstrated to promote EMT and osteosarcoma progression by inhibiting ATP5A1 ubiquitination-dependent degradation and activating downstream AKT/mTOR signaling. These findings offer compelling evidence that HSPD1 is a promising prognostic biomarker and therapeutic target for osteosarcoma.

## Supplementary Material

Supplementary materials and methods, figures and tables.

## Abbreviations

HSPs: heat shock proteins
NOA: non-oncogene addiction
TME: tumor microenvironment
HCC: hepatocellular carcinoma
OXPHOS: oxidative phosphorylation
qRT-PCR: quantitative real-time reverse transcription-PCR
CCK-8: cell counting kit-8
WB: Western blotting
Co-IP: coimmunoprecipitation
CHX: cycloheximide
HE: Haematoxylin-eosin
IHC: immunohistochemistry
scRNA-seq: single-cell RNA sequencing
TCGA: The Cancer Genome Atlas
GSEA: gene set enrichment analysis
GSVA: gene set variation analysis
ssGSEA: single-sample GSEA
OS: overall survival
CAFs: cancer-associated fibroblasts
DCs: dendritic cells
pDCs: plasmacytoid dendritic cells
TILs: tumor-infiltrating lymphocytes
NK cells: natural killer cells
Tfh cells: T follicular helper cells
Tregs: regulatory T cells
CCR: cytokine-cytokine receptor
HLA: human leukocyte antigen
ICGs: immune checkpoint genes
MHC: major histocompatibility complex
coIP/MS: co-immunoprecipitation/mass spectrometry
PPI: protein-protein interaction
ICIs: immune checkpoint inhibitors
ICB: immune checkpoint blockade
EMT: epithelial-mesenchymal transition
CRC: colorectal cancer
p-AKT: phosphorylated AKT
p-mTOR: phosphorylated mTOR
ATP: adenosine triphosphate
BKA: Bongkrekic acid
ANT: adenine nucleotide translocator
TIME: tumor-immune microenvironment
