# Supplementary material for: HSPD1 Supports Osteosarcoma Progression through Stabilizing ATP5A1 and thus Activation of AKT/mTOR Signaling
Source: Int J Biol Sci. 2024 Sep 23;20(13):5162–90. doi: 10.7150/ijbs.100015 (PMC11489178; doi:10.7150/ijbs.100015)
Supplement: Supplementary file 1 — Supplementary materials and methods, figures and tables. [file ijbsv20p5162s1.pdf]

HSPD1 Supports Osteosarcoma Progression through Stabilizing ATP5A1 and thus Activation of AKT/mTOR Signaling

Yiming Zhang[1†], Ruilin Pan[1†], Kun Li[1,2†], Lek Hang Cheang[3†], Jing Zhao[4], Zhangfeng Zhong[4], Shaoping Li[4], Jinghao Wang[5,6], Xiaofang Zhang[5,7], Yanmei Cheng[8*], Xiaofei Zheng[1*], Rongrong He[2*], and Huajun Wang[1*]

[1]Department of Sports Medicine, The First Affiliated Hospital, Guangdong Provincial Key Laboratory of Speed Capability, The Guangzhou Key Laboratory of Precision Orthopedics and Regenerative Medicine, Jinan University, Guangzhou, China

[2]State Key Laboratory of Bioactive Molecules and Drug Ability Assessment, Guangdong Engineering Research Center of Chinese Medicine & Disease Susceptibility, International Cooperative Laboratory of Traditional Chinese Medicine Modernization and Innovative Drug Development of the Chinese Ministry of Education, Guangdong Province Key Laboratory of Pharmacodynamic Constituents of Traditional Chinese Medicine and New Drugs Research, Jinan University, Guangzhou, China.

[3]Department of Orthopedic Surgery, Centro Hospitalar Conde de Sao Januario, Macau, China

[4]State Key Laboratory of Quality Research in Chinese Medicine, Institute of Chinese Medical Sciences, Department of Pharmaceutical Sciences, Faculty of Health Sciences, University of Macau, China

[5]Department of Pharmacy, the First Affiliated Hospital, State Key Laboratory of Frigid Zone Cardiovascular Diseases, Jinan University, Guangzhou, China.

[6]Department of Orthopedics, NHC Key Laboratory of Cell Transplantation, The First Affiliated Hospital of Harbin Medical University, Harbin, China

[7]Department of Pharmacology (State-Province Key Laboratories of Biomedicine-Pharmaceutics of China, Key Laboratory of Cardiovascular Research, Ministry of Education), College of Pharmacy, Harbin Medical University, Harbin, 150086, Heilongjiang, China.

[8]Department of Cardiothoracic Surgery ICU, The First Affiliated Hospital of Sun Yat-sen University,Guangzhou, 510080, Guangdong, China.

[†] These authors have contributed equally to this work.

This content includes:

1) Supplementary Materials and Methods

2) Nine supplementary figures

3) Three supplementary tables

Supplementary Materials and Methods

Chemicals and reagents

The AKT activator SC79 and AKT inhibitor MK2206 were purchased from MedChemExpress (Shanghai, China). The working concentration of SC79 and MK2206 was 5 μM.

Antibodies regents for western blotting

The following primary antibodies were utilized in this study: anti-HSPD1 (1:8000, 15282-1-AP, Proteintech, Wuhan, China), anti-E-cadherin (1:5000, 60335-1-Ig, Proteintech, Wuhan, China), anti-N-cadherin (1:8000, 22018-1-AP, Proteintech, Wuhan, China), anti-Vimentin (1:6000, 10366-1-AP, Proteintech, Wuhan, China), anti-mTOR (1:10000, 66888-1-Ig, Proteintech, Wuhan, China), anti-p-mTOR (1:6000, 67778-1-Ig, Proteintech, Wuhan, China), anti-AKT (1:10000, 60203-2-Ig, Proteintech, Wuhan, China), anti-p-AKT (1:6000, 66444-1-Ig, Proteintech, Wuhan, China), anti-ATP5A1 (1:10000, 14676-1-AP, Proteintech, Wuhan, China), anti-ubiquitin (1:1000, 10201-2-AP, Proteintech, Wuhan, China), anti-Flag (1:10000, 66008-4-Ig, Proteintech, Wuhan, China), anti-HA (1:10000, 66006-2-Ig, Proteintech, Wuhan, China), anti-K48Ub (1:1000, ab140601, Abcam, Shanghai, China), and anti-β-actin (1:7000, 20536-1-AP, Proteintech, Wuhan, China). The following secondary antibodies were utilized in this study: Horseradish peroxidase (HRP)-conjugated AffiniPure goat anti-rabbit IgG (H+L) (1:5000, SA00001-2, Proteintech, Wuhan, China) and HRP-conjugated AffiniPure goat anti-mouse IgG (H+L) (1:5000, SA00001-1, Proteintech, Wuhan, China).

Wound healing and transwell assay

Transwell plates (24 wells, 8 μm pore size, Corning) were used for the transwell assay. 1×10^5 osteosarcoma cells were collected with 200 μL serum-free medium and added to the upper chamber without or with matrix. Then 600 μL of 10% FBS-containing medium was added to the lower chamber as a chemoattractant. After 24 hours of incubation, cells passing through the insert were fixed with 4% paraformaldehyde and stained with crystal violet. The migrated cells were photographed and counted under an inverted microscope. For the scratch assay, linear scratching was performed using 200μl pipette tips, and cell debris was removed using PBS washing. Cells were then supplemented with 2 ml serum-free medium. Wound width was recorded every 24 hours by inverted microscopy and images were analyzed using ImageJ. Specifically, wound closure was quantified using the line tool in ImageJ software to measure the wound margin and the wound area. The healing rate was calculated as follows: Healing rate = (Initial wound area - non-healing area)/initial wound area.

LC-MS/MS analysis for quantitative proteomics

Cell lysates were centrifuged at 12,000 rpm for 15 minutes and the supernatant was mixed with HSPD1 antibody on a 4°C shaker overnight and then incubated with pre-washed protein A/G agarose beads for 4 hours. The bound proteins were eluted with Laemmli buffer containing 500 µL of 6 M urea, 25 µL of 100 mM DTT, and 25 µL of 400 mM IAA for 30 minutes at 25°C under dark conditions. The eluate was incubated with 150 µL of 2 M urea, 150 µL of 1 mMCaCl2, and 10 µg of trypsin at 37°C overnight. Afterward, peptide samples were desalted using MonoTip C18 (Shimadzu Biotech, Japan) and analyzed by LC-MS/MS (HPLC system coupled to a Q-Exactive Plus mass spectrometer, Thermo Scientific, Germany). Proteins were identified by searching against the human proteome database (uniprotkb_AND_model_organism_9606_AND_r_2024_03_09.fasta) downloaded from UniProt and were quantified with the label-free quantitative (LFQ) algorithm embedded in MaxQuant version 2.4.14.0. After MaxQuant analysis, the iBAQ values were normalized to the total iBAQ sum. The protein group files were imported into Perseus software (version 2.0.11) to perform statistical analysis and validation. For the calculation of enriched proteins in the experimental group (HSPD1 antibody) versus controls (IgG antibody), only proteins with two or more unique peptides and a P value < 0.05 using a two-tailed Student's t-test were considered. Protein-protein interaction (PPI) network of differentially expressed proteins was generated by STRING version 12.0 (http://string-db.org/).

Hematoxylin-eosin (HE) and immunohistochemistry (IHC) staining

Formalin-fixed, paraffin-embedded tumors were deparaffinized in xylene and rehydrated sequentially in ethanol. For HE staining, sectioned tumors were stained with hematoxylin and eosin. For IHC, tumor sections were incubated with primary antibodies at 4°C overnight after rehydration, antigen retrieval, and sealing. The following primary antibodies were used: anti-HSPD1 (1:300, 15282-1-AP, Proteintech, Wuhan, China), anti-ATP5A1 (1:300, 14676-1-AP, Proteintech, Wuhan, China), anti-E-cadherin (1:1000, 60335-1-Ig, Proteintech, Wuhan, China), anti-N-cadherin (1:4000, 22018-1-AP, Proteintech, Wuhan, China), anti-Ki67 (1:500, GB151499-100, Service Bio, Wuhan, China), and anti-Vimentin (1:5000, 10366-1-AP, Proteintech, Wuhan, China). Images of all tumor sections were captured using Pannoramic 250 Flash (3DHISTECH Ltd., Budapest, Hungary).

Consensus clustering analysis to identify HSP molecular subtypes of osteosarcoma

Univariate Cox regression was performed to screen for prognostic HSPs based on gene lists from previous studies using the "survival" package. A hazard ratio (HR) greater than 1 indicates a worse prognosis and vice versa. Unsupervised consensus clustering based on the K-means clustering algorithm was used to identify potential HSP modification patterns in osteosarcoma via the "ConsensusClusterPlus" package. We set the cluster number (k) between two and ten and confirmed the optimal cluster count using cumulative distribution function (CDF) and consensus

matrices. Kaplan-Meyer (KM) analysis was performed relying on the “survival” and “survminer” packages to estimate the overall survival (OS) of diverse HSP molecular subtypes. PCA analysis was performed to verify the heterogeneity between distinct HSP phenotypes. Finally, the expression levels of prognostically relevant HSPs in different HSP-based subtypes were analyzed using the "limma" package and displayed as box plots and heat maps.

Functional enrichment analysis of HSP molecular subtypes

We used marker gene sets (c2.cp.kegg_medicus.v2023.2.Hs.symbols.gmt) for gene set variation analysis (GSVA) to understand the specific functions and enrichment pathways of different molecular subtypes. We performed gene set enrichment analysis (GSEA) using marker gene sets (c6.all.v2023.2.Hs.symbols.gmt) to understand oncogenic signaling in different molecular subtypes. An adjusted P value < 0.05 was considered statistically significant.

Construction of the HSP-based risk stratification system

Based on prognostically relevant HSPs, least absolute shrinkage and selection operator regression (LASSO) and multivariate Cox regression were used to further screen key HSPs and refine the HSP scoring system. The HSPscores = $\Sigma(coefi \times Expi)$, where coefi is the coefficient of each gene in the HSP scoring system and Expi is HSP gene expression. The osteosarcoma meta-cohort was randomized 1:1 into training and test groups using the "caret" package. KM survival curves were used to compare the OS time of patients in different HSP scoring groups. Receiver operating characteristic (ROC) curves were plotted using the "timeROC" package to estimate the predictive accuracy of HSPscores in the train, test, and entire cohorts.

Tumor microenvironment and drug sensitivity analysis

The ESTIMATE algorithm was employed to calculate tumor purity, immune score, and stromal score for each sample. The activity of immune-related pathways and the abundance of tumor-infiltrating immune cells were calculated for each osteosarcoma sample using the single-sample gene set enrichment analysis (ssGSEA) algorithm utilizing the “GSEABase” and “GSVA” packages. In addition, immune checkpoint genes (ICGs) and major histocompatibility complex (MHC) molecules were evaluated in different HSP scoring subgroups, and the Wilcoxon rank sum test was used to resolve differences between the two groups. The IC 50 for each drug in individual osteosarcoma patients was estimated based on the Genomics of Drug Sensitivity in Cancer (GDSC, https://www.cancerrxgene.org) via the "oncoPredict" package.

Clinical correlation analysis and comparative analysis

COX regression was used to determine whether the HSPscore was an independent prognostic factor. Correlations between HSPscore and clinicopathologic characteristics were analyzed using Kruskal-Wallis and Wilcoxon rank-sum tests. KM

survival analyses were performed in different subgroups according to age (≤18 and >18 years), sex (female and male), and metastatic status (metastatic and non-metastatic). We named the published prognostic scoring systems as Yang [1], Han [2], Zhang [3], and Jin [4] signatures according to the authors' names, and then compared them with the HSPscore in robustness using the "survival", "survival", and "timeROC" packages.

Subcellular localization analysis and single-cell RNA-Seq analysis

The Human Protein Atlas (HPA, https://www.Proteinatlas.org/) and PDB database (https://www.rcsb.org/) helped determine the subcellular localization and protein structure of the core HSPs, respectively. In addition, single-cell RNA-seq (scRNA-seq) data from the GSE162454 cohort were mined using TISCH2 (http://tisch.comp-genomics.org/), and single-cell HSPD1 mRNA expression in immune-infiltrating and osteosarcoma cells was assessed after eliminating inter-sample batches, uniformly annotating cell types, and identifying malignant cells.

Differential expression analysis between high and low HSPD1 expression groups

Differential expression analysis between high and low HSPD1 expression groups was analyzed using the "limma" package with false discovery rate (FDR) < 0.05. Gene Ontology (GO) and Kyoto Encyclopedia of Genes and Genomes (KEGG) pathway analyses were then performed using the "clusterProfiler" package.

Graphical abstract

The graphical abstract was conducted by Figdraw.

Reference

1. Yang W, Wu H, Tong L, Wang Y, Guo Q, Xu L, et al. A cuproptosis-related genes signature associated with prognosis and immune cell infiltration in osteosarcoma. Frontiers in oncology. 2022; 12: 1015094.
2. Han S, Wang Q, Shen M, Zhang X, Wang J. Immunogenic cell death related mRNAs associated signature to predict immunotherapeutic response in osteosarcoma. Heliyon. 2024; 10: e27630.
3. Zhang Y, He R, Lei X, Mao L, Jiang P, Ni C, et al. A Novel Pyroptosis-Related Signature for Predicting Prognosis and Indicating Immune Microenvironment Features in Osteosarcoma. Frontiers in genetics. 2021; 12: 780780.
4. Jin Z, Wu J, Lin J, Wang J, Shen Y. Identification of the Transcription Co-Factor-Related Gene Signature and Risk Score Model for Osteosarcoma. Frontiers in genetics. 2022; 13: 862803.

Supplementary Figures

Figure S1

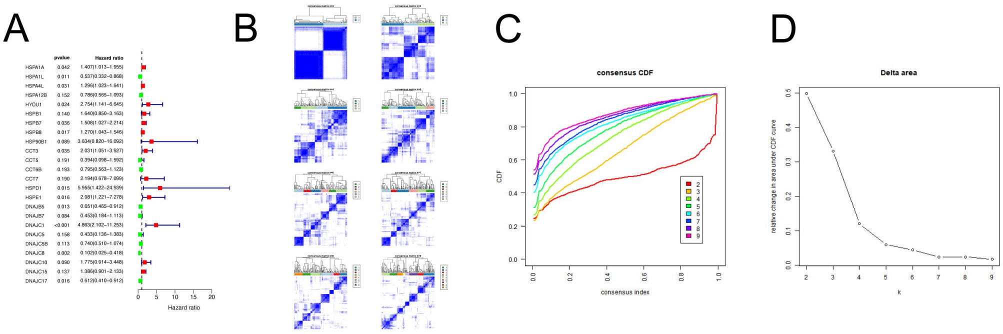

Figure S1. Unsupervised clustering of prognostic HSPs in the osteosarcoma meta-cohort. (A) Prognostic HSPs in osteosarcoma filtered by univariate Cox regression. **(B)** Unsupervised clustering of 24 prognostic HSPs in osteosarcoma cohort and consensus matrices for k=2-9. **(C)** The cumulative distribution function plot depicting the cumulative distribution from consensus matrices at a given cluster number (k). **(D)** the delta plot assessing change in the CDF area.

Figure S2

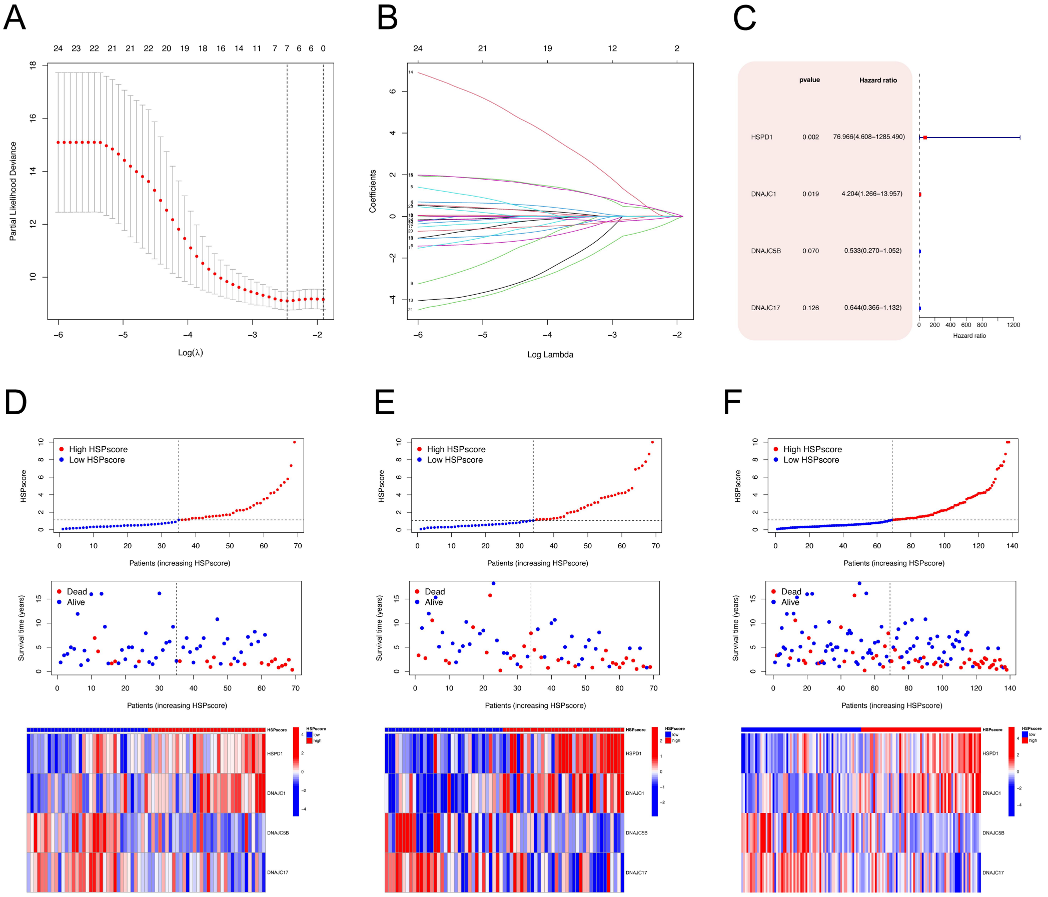

Figure S2. Prognostic HSPs were screened using LASSO regression and multivariate Cox analysis. (A) Identification of the best parameter (λ) in the LASSO. **(B)** LASSO coefficient profiles of each variable against the log(λ). **(C)** Four critical HSPs (HSPD1, DNAJC1, DNAJC5B, and DNAJC17) were identified under multiCox analysis. **(D)** Distribution of HSPscores, overall survival status, and expression of four critical HSPs in the training cohort. **(E)** Distribution of HSPscores, overall survival status, and expression of four critical HSPs in the testing groups. **(F)** Distribution of HSPscores, overall survival status, and expression of four critical HSPs in the entire cohort.

Figure S3

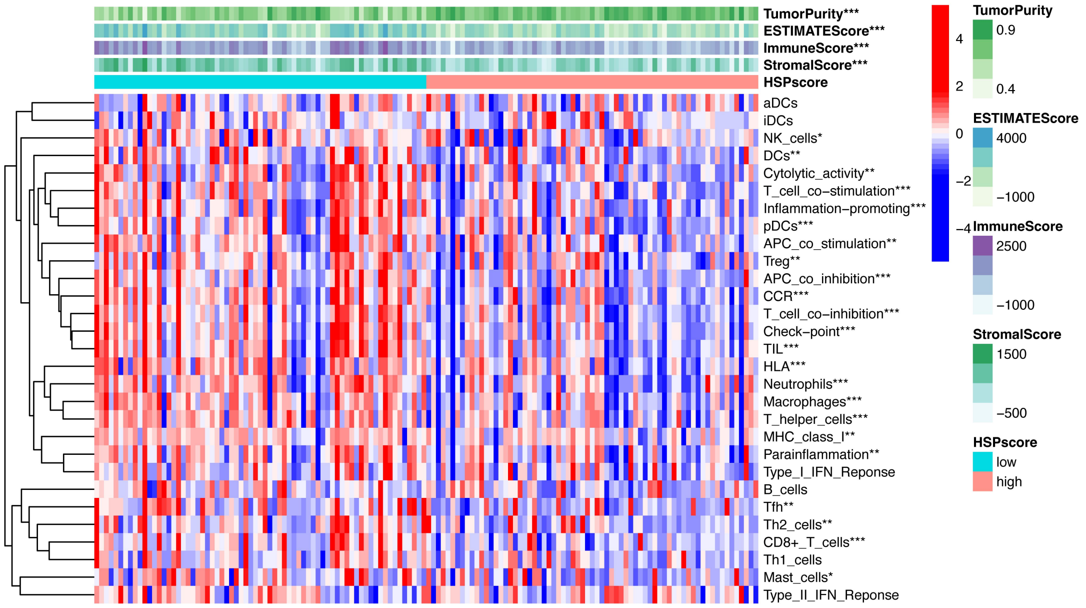

Figure S3. Heatmap for visualization of differences in the immune score, stromal score, ESTIMATE score, tumor purity, immune cells, and immune functions between diverse HSPscore subgroups.

Figure S4

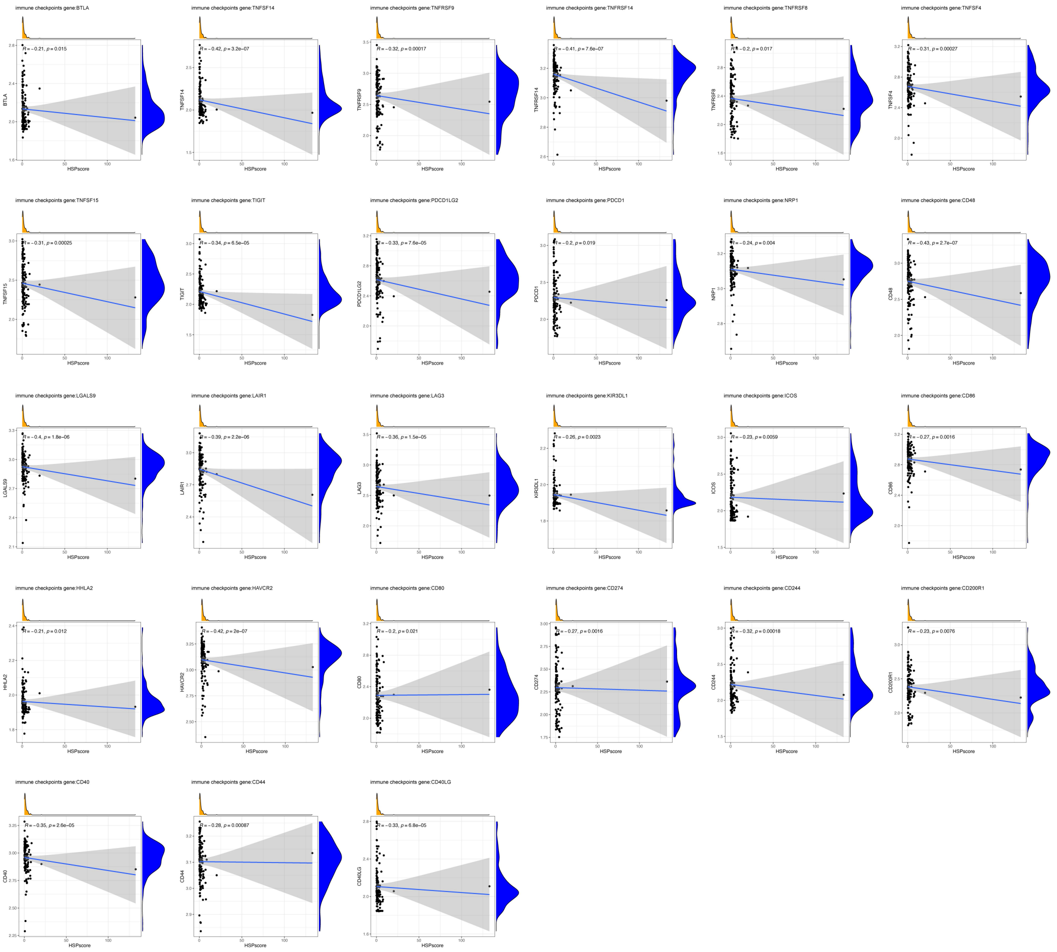

Figure S4. The correlation between HSPscore and immune checkpoints in osteosarcoma.

Figure S5

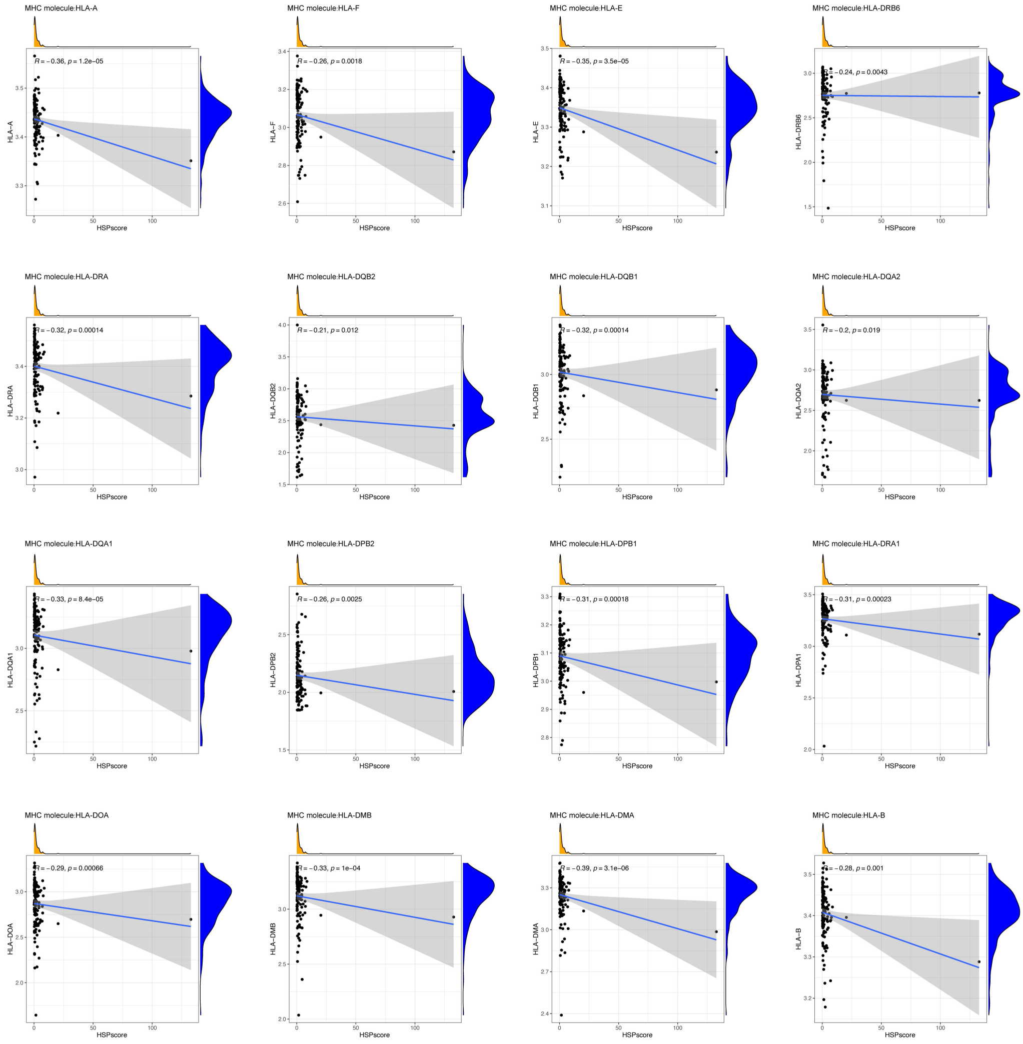

Figure S5. The correlation between HSPscore and major histocompatibility complex (MHC) molecules in osteosarcoma.

Figure S6

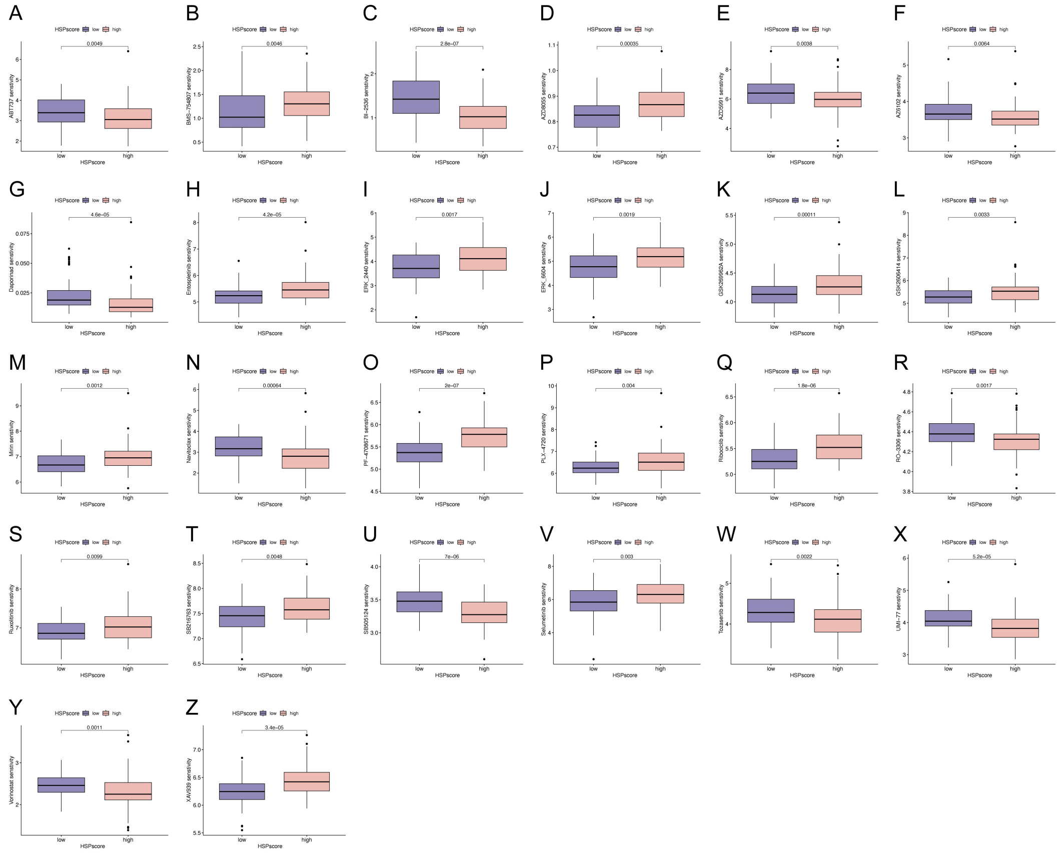

Figure S6. Discrepancies in drug sensitivity between diverse HSPscore subgroups for each compound from the Genomics of Drug Sensitivity in Cancer database, including ABT737 (A), BMS-754807 (B), BI-2536 (C), AZD8055 (D), AZD5991 (E), AZ6102 (F), Daporinad (G), Entospletinib (H), ERK 2440 (I), ERK 6604 (J), GSK269962A (K), GSK2606414 (L), Mirin (M), Navitoclax (N), PF-4708671 (O), PLX-4720 (P), Ribociclib (Q), RO-3306 (R), Ruxolitinib (S), SB216763 (T), SB505124 (U), Selumetinib (V), Tozasertib (W), UMI-77 (X), Vorinostat (Y), and XAV939 (Z).

Figure S7

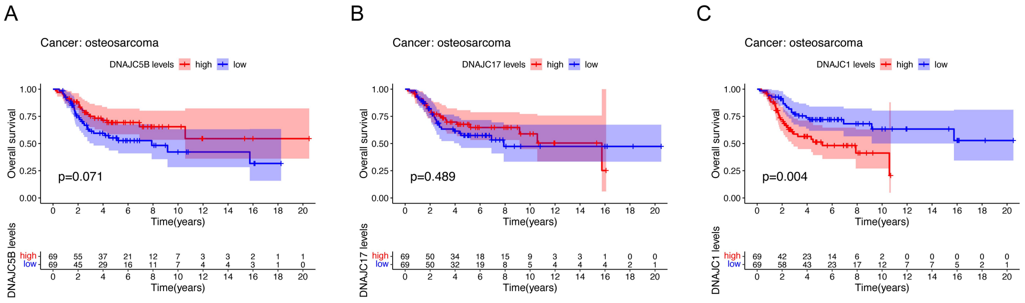

Figure S7. Kaplan-Meier survival analysis of osteosarcoma patients stratified by DNAJC1 (A), DNAJC5B (B), and DNAJC17 (C) expression levels.

Figure S8

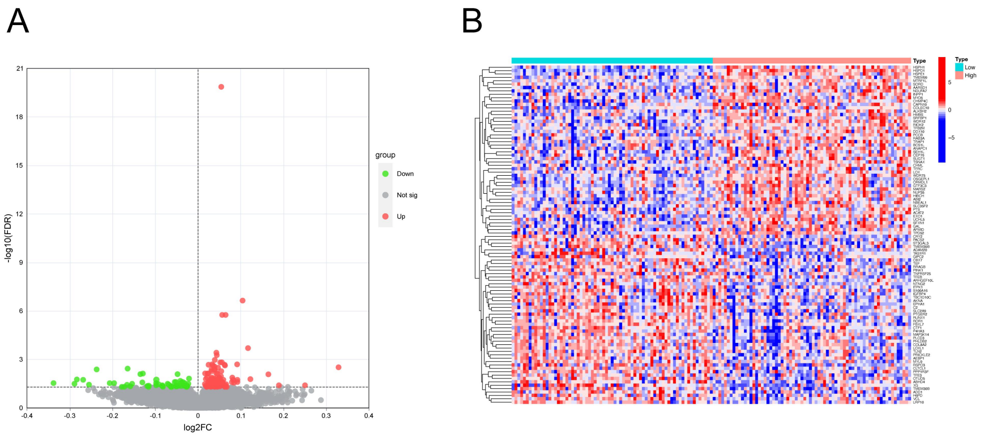

Figure S8. Differential expression analysis between high and low HSPD1 expression groups. (A) A volcano plot shows differentially expressed genes between the high and low HSPD1 expression groups. **(B)** A heatmap shows 50 up-regulated genes and 50 down-regulated genes with the largest differential changes.

Figure S9

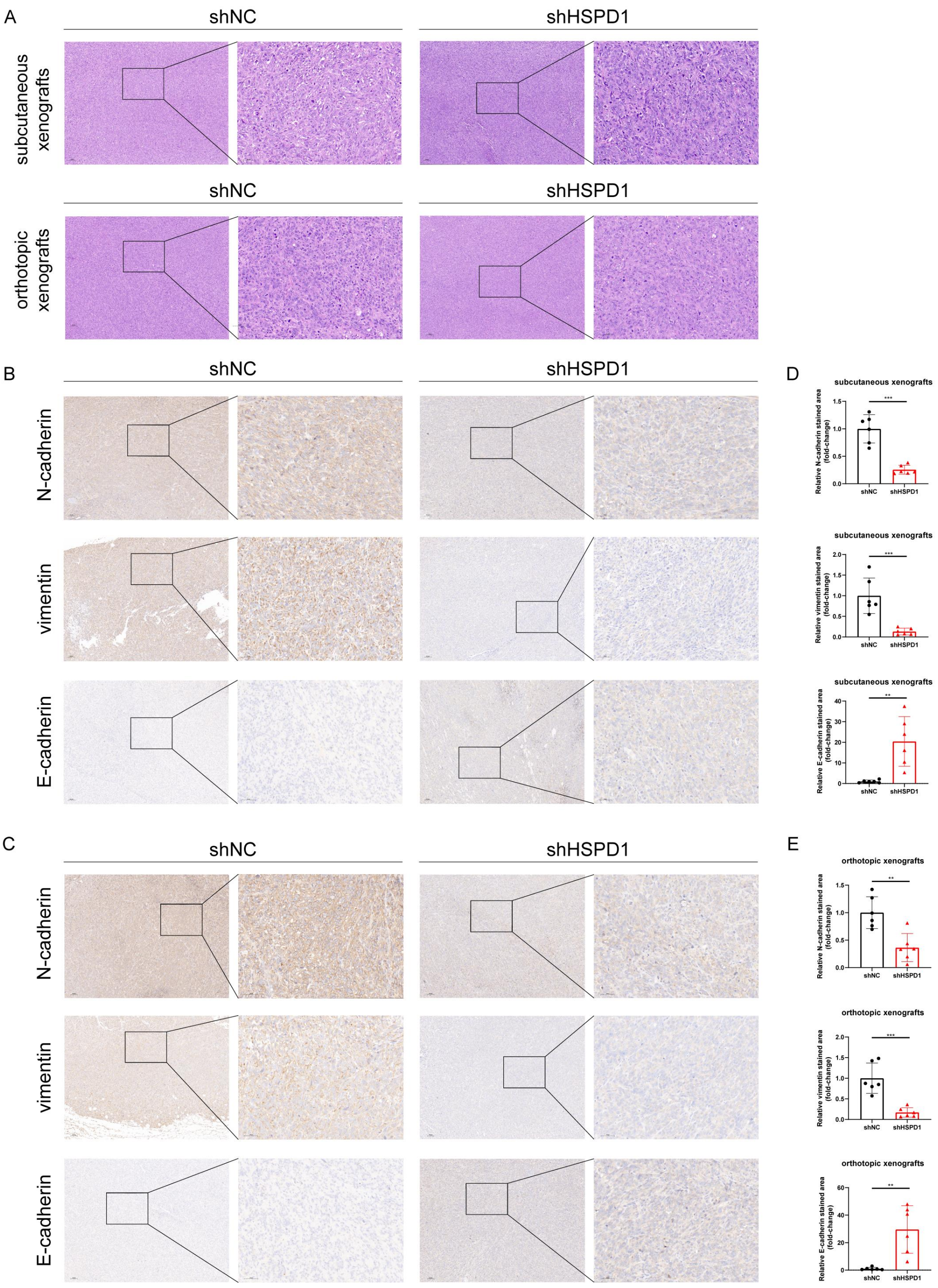

Figure 9. HSPD1 depletion impairs the epithelial-mesenchymal transition of osteosarcoma cells in vivo. (A) Hematoxylin and eosin (HE) staining in subcutaneous and orthotopic xenograft tumors derived from the shNC and shHSPD1 groups. Montage scale bar, 100μm; magnified-view scale bar, 50μm. **(B)** Immunohistochemical (IHC) analysis of N-cadherin, vimentin, and E-cadherin in subcutaneous xenograft tumors derived from the shNC and shHSPD1 groups. Montage scale bar, 100μm; magnified-view scale bar, 50μm. **(C)** IHC analysis of N-cadherin, vimentin, and E-cadherin in orthotopic xenograft tumors derived from the

shNC and shHSPD1 groups. Montage scale bar, 100μm; magnified-view scale bar, 50μm. **(D)** Semiquantitative analysis of IHC staining for N-cadherin, vimentin, and E-cadherin in subcutaneous xenograft models of osteosarcoma. **(E)** Semiquantitative analysis of IHC staining for N-cadherin, vimentin, and E-cadherin in orthotopic xenograft models of osteosarcoma. The data are presented as mean ± SD. $^{*}P < 0.05$; $^{**}P < 0.01$; $^{***}P < 0.001$.

Supplementary Tables

Supplementary Table S1. Primer sequences used in the study.

	**Forward Primer (5′→3′)**	**Reverse Primer (5′→3′)**
HSPD1	CGCGCTCAACATGCACCTA	GCAGTAGAATTTCGGTCCAGTT
ATP5A1	CTCCAGATTATGCCTATGGTGC	AGCTCCACATCGAAGACGAGA
β-actin	CATGTACGTTGCTATCCAGGC	CTCCTTAATGTCACGCACGAT

Supplementary Table S2. The shRNA sequences targeting the human HSPD1 gene and ATP5A1 gene in the study as well as the negative control (NC) sequences.

	sequence (5′→3′)
shHSPD1#1	GTTGCAAAGTCAATTGACT
shHSPD1#2	GTTGCTACGATTTCTGCAA
shATP5A1#1	TCTGCTTACATTCCAACAAAT
shATP5A1#2	CGTTTCAATGATGGATCTGAT
shNC	UUCUCCGAACGUGUCACGUTT

Supplementary Table S3. List of 95 HSP family members. For each HSP, the gene name, protein name, and Uniprot database access number are reported.

**Gene Name**	**Protein Name**	**Uniprot Access**
HSPA1A	Heat shock 70 kDa protein 1A	P0DMV8
HSPA1B	Heat shock 70 kDa protein 1B	P0DMV9
HSPA1L	Heat shock 70 kDa protein 1-like	P34931
HSPA2	Heat shock-related 70 kDa protein 2	P54652
HSPA4	Heat shock 70 kDa protein 4	P34932
HSPA4L	Heat shock 70 kDa protein 4L	O95757
HSPA5	Endoplasmic reticulum chaperone BiP	P11021
HSPA6	Heat shock 70 kDa protein 6	P17066
HSPA7	Putative heat shock 70 kDa protein 7	P48741
HSPA8	Heat shock cognate 71 kDa protein	P11142
HSPA9	Stress-70 protein, mitochondrial	P38646
HSPA12A	Heat shock 70 kDa protein 12A	O43301
HSPA12B	Heat shock 70 kDa protein 12B	Q96MM6
HSPA13	Heat shock 70 kDa protein 13	P48723
HSPA14	Heat shock 70 kDa protein 14	Q0VDF9

HSPH1	Heat shock protein 105 kDa	Q92598
HYOU1	Hypoxia up-regulated protein 1	Q9Y4L1
HSPB1	Heat shock protein beta-1	P04792
HSPB2	Heat shock protein beta-2	Q16082
HSPB3	Heat shock protein beta-3	Q12988
HSPB4/CRYAA	Alpha-crystallin A chain	P02489
HSPB5/CRYAB	Alpha-crystallin B chain	P02511
HSPB6	Heat shock protein beta-6	O14558
HSPB7	Heat shock protein beta-7	Q9UBY9
HSPB8	Heat shock protein beta-8	Q9UJY1
HSPB9	Heat shock protein beta-9	Q9BQS6
HSPB10/OFD1	Oral-facial-digital syndrome 1 protein	O75665
HSPB11	Intraflagellar transport protein 25 homolog	Q9Y547
HSP90AA1	Heat shock protein HSP 90-alpha	P07900
HSP90AB1	Heat shock protein HSP 90-beta	P08238
HSP90B1	Endoplasmin	P14625
HSP90L/TRAP1	Heat shock protein 75 kDa, mitochondrial	Q12931
BBS10	Bardet-Biedl syndrome 10 protein	Q8TAM1
BBS12	Bardet-Biedl syndrome 12 protein	Q6ZW61
CCT1/TCP1	T-complex protein 1 subunit alpha	P17987
CCT2	T-complex protein 1 subunit beta	P78371
CCT3	T-complex protein 1 subunit gamma	P49368
CCT4	T-complex protein 1 subunit delta	P50991
CCT5	T-complex protein 1 subunit epsilon	P48643
CCT6A	T-complex protein 1 subunit zeta	P40227
CCT6B	T-complex protein 1 subunit zeta-2	Q92526
CCT7	T-complex protein 1 subunit eta	Q99832
CCT8	T-complex protein 1 subunit theta	P50990
HSPD1	60 kDa heat shock protein, mitochondrial	P10809
HSPE1	10 kDa heat shock protein, mitochondrial	P61604
MKKS	McKusick-Kaufman/Bardet-Biedl syndromes putative chaperonin	Q9NPJ1
DNAJA1	DnaJ homolog subfamily A member 1	P31689
DNAJA2	DnaJ homolog subfamily A member 2	O60884
DNAJA3	DnaJ homolog subfamily A member 3, mitochondrial	Q96EY1
DNAJA4	DnaJ homolog subfamily A member 4	Q8WW22
DNAJB1	DnaJ homolog subfamily B member 1	P25685
DNAJB2	DnaJ homolog subfamily B member 2	P25686
DNAJB3	DnaJ homolog subfamily B member 3	Q8WWF6
DNAJB4	DnaJ homolog subfamily B member 4	Q9UDY4
DNAJB5	DnaJ homolog subfamily B member 5	O75953
DNAJB6	DnaJ homolog subfamily B member 6	O75190
DNAJB7	DnaJ homolog subfamily B member 7	Q7Z6W7
DNAJB8	DnaJ homolog subfamily B member 8	Q8NHS0

DNAJB9	DnaJ homolog subfamily B member 9	Q9UBS3
DNAJB11	DnaJ homolog subfamily B member 11	Q9UBS4
DNAJB12	DnaJ homolog subfamily B member 12	Q9NXW2
DNAJB13	DnaJ homolog subfamily B member 13	P59910
DNAJB14	DnaJ homolog subfamily B member 14	Q8TBM8
DNAJC1	DnaJ homolog subfamily C member 1	Q96KC8
DNAJC2	DnaJ homolog subfamily C member 2	Q99543
DNAJC3	DnaJ homolog subfamily C member 3	Q13217
DNAJC4	DnaJ homolog subfamily C member 4	Q9NNZ3
DNAJC5	DnaJ homolog subfamily C member 5	Q9H3Z4
DNAJC5B	DnaJ homolog subfamily C member 5B	Q9UF47
DNAJC5G	DnaJ homolog subfamily C member 5G	Q8N7S2
DNAJC6	Putative tyrosine-protein phosphatase auxilin	O75061
DNAJC7	DnaJ homolog subfamily C member 7	Q99615
DNAJC8	DnaJ homolog subfamily C member 8	O75937
DNAJC9	DnaJ homolog subfamily C member 9	Q8WXX5
DNAJC10	DnaJ homolog subfamily C member 10	Q8IXB1
DNAJC11	DnaJ homolog subfamily C member 11	Q9NVH1
DNAJC12	DnaJ homolog subfamily C member 12	Q9UKB3
DNAJC13	DnaJ homolog subfamily C member 13	O75165
DNAJC14	DnaJ homolog subfamily C member 14	Q6Y2X3
DNAJC15	DnaJ homolog subfamily C member 15	Q9Y5T4
DNAJC16	DnaJ homolog subfamily C member 16	Q9Y2G8
DNAJC17	DnaJ homolog subfamily C member 17	Q9NVM6
DNAJC18	DnaJ homolog subfamily C member 18	Q9H819
DNAJC19	Mitochondrial import inner membrane translocase subunit TIM14	Q96DA6
DNAJC20/HSCB	Iron-sulfur cluster co-chaperone protein HscB	Q8IWL3
DNAJC21	DnaJ homolog subfamily C member 21	Q5F1R6
DNAJC22	DnaJ homolog subfamily C member 22	Q8N4W6
DNAJC23/SEC63	Translocation protein SEC63 homolog	Q9UGP8
DNAJC24	DnaJ homolog subfamily C member 24	Q6P3W2
DNAJC25	DnaJ homolog subfamily C member 25	Q9H1X3
DNAJC26/GAK	Cyclin-G-associated kinase	O14976
DNAJC27	DnaJ homolog subfamily C member 27	Q9NZQ0
DNAJC28	DnaJ homolog subfamily C member 28	Q9NX36
DNAJC29/SACS	Sacsin	Q9NZJ4
DNAJC30	DnaJ homolog subfamily C member 30, mitochondrial	Q96LL9
